# Novel thermoplastic microvalves based on an elastomeric cyclic olefin copolymer†

**DOI:** 10.1039/d4lc00501e

**Published:** 2024-08-13

**Authors:** Katie Childers, Ian M. Freed, Mateusz L. Hupert, Benjamin Shaw, Noah Larsen, Paul Herring, Jeanne H. Norton, Farhad Shiri, Judy Vun, Keith J. August, Małgorzata A. Witek, Steven A. Soper

**Affiliations:** a Bioengineering Program, The University of Kansas Lawrence KS 66045 USA ssoper@ku.edu; b Center of BioModular Multiscale Systems for Precision Medicine, The University of Kansas Lawrence KS 66045 USA; c Department of Chemistry, The University of Kansas Lawrence KS 66045 USA; d BioFluidica Inc. San Diego CA 92121 USA; e Department of Chemical Engineering, The University of Kansas Lawrence KS 66045 USA; f Department of Engineering Physics, The University of Kansas Lawrence KS 66045 USA; g Department of Plastics Engineering Technology, Pittsburg State University Pittsburg KS 66762 USA; h Department of Pediatrics, Children's Mercy Hospital Kansas City MO 64108 USA; i Department of Mechanical Engineering, The University of Kansas Lawrence KS 66045 USA; j KU Cancer Center, University of Kansas Medical Center Kansas City KS 66160 USA

## Abstract

Microfluidic systems combine multiple processing steps and components to perform complex assays in an autonomous fashion. To enable the integration of several bio-analytical processing steps into a single system, valving is used as a component that directs fluids and controls introduction of sample and reagents. While elastomer polydimethylsiloxane has been the material of choice for valving, it does not scale well to accommodate disposable integrated systems where inexpensive and fast production is needed. As an alternative to polydimethylsiloxane, we introduce a membrane made of thermoplastic elastomeric cyclic olefin copolymer (eCOC), that displays unique attributes for the fabrication of reliable valving. The eCOC membrane can be extruded or injection molded to allow for high scale production of inexpensive valves. Normally hydrophobic, eCOC can be activated with UV/ozone to produce a stable hydrophilic monolayer. Valves are assembled following *in situ* UV/ozone activation of eCOC membrane and thermoplastic valve seat and bonded by lamination at room temperature. eCOC formed strong bonding with polycarbonate (PC) and polyethylene terephthalate glycol (PETG) able to hold high fluidic pressures of 75 kPa and 350 kPa, respectively. We characterized the eCOC valves with mechanical and pneumatic actuation and found the valves could be reproducibly actuated >50 times without failure. Finally, an integrated system with eCOC valves was employed to detect minimal residual disease (MRD) from a blood sample of a pediatric acute lymphoblastic leukemia (ALL) patient. The two module integrated system evaluated MRD by affinity-selecting CD19(+) cells and enumerating leukemia cells *via* immunophenotyping with ALL-specific markers.

## Abstract 2

We introduce cyclic olefin copolymer elastomer as a membrane layer for pneumatically or mechanically actuated microvalves using a simple bonding procedure and demonstrate its utility within a modular system for CLC isolation and immunophenotyping.

## Graphical abstract

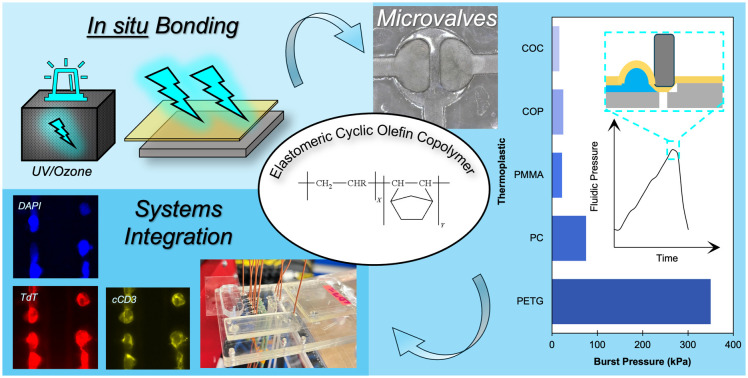

## 1. Introduction

Current medical laboratory tests require highly specialized equipment and trained operators, which can be expensive, prone to errors, and increase the “sample-to-answer” time providing delayed diagnosis. In contrast to conventional benchtop laboratory methods and to better facilitate the decentralization of medical testing into a variety of clinical settings that traditionally are not well equipped to carry out highly specialized medical tests, development of *in vitro* diagnostic (IVD) tests based on the use of microfluidics aims to reduce time, cost, and resources needed for disease management primarily by providing full process automation of the IVD test. IVD-based microfluidic tools can utilize paper microfluidics to perform lateral flow assays that evaluate the presence or absence of a single marker.^1^ However, as the assay and biomarker analyses become more demanding, the required tools must employ more technically advanced tactics to provide favorable analytical and clinical figures-of-merit. An effective decentralized IVD should include: (i) accessible biomarkers that have high diagnostic value; (ii) simple instrumentation that provides sample processing automation; and (iii) minimal demands on operators in terms of high degree of training to carry out the required test.

To realize IVD for advanced and complex bioassays requiring multiple processing steps, integrated fluidic systems combining multiple components (*i.e.*, devices) on a single autonomous platform are envisioned. Devices perform a single task such as biomarker isolation from a complex clinical sample,^2^ enumeration,^3^ amplification,^4^ or electrokinetic separations.^5,6^ The system must perform unit operations as well, such as heating/cooling, fluid control and mixing, connections, or light exposure. Integrated systems can be realized in primarily three different configurations including centrifugal microfluidics,^7–9^ monolithic,^10–12^ or modular systems. We have reported on several different modular systems that use task-specific modules and a fluidic motherboard with the appropriate interconnects for a variety of applications including infectious diseases,^13^ and the analysis of liquid biopsy markers, such as circulating tumor cells.^14^

To allow integrated microfluidic systems, independent of their configuration, to run without user intervention, valves must be included to control fluidic flow of samples and/or reagents. There are various microfluidic valves that have been reported^15–17^ including capillary pressure control valves,^18^ burst valves,^19,20^ pneumatic actuation,^21^ magnetic actuation,^22,23^ and thermal actuation.^24,25^ To realize the successful development of IVD-based microfluidic systems, cost and ease of operation are important factors in valve choice, both in terms of actuation mode and the material used for the valving operation. In addition, process yield rates are critically important, especially in integrated systems, to keep assay cost low. Membrane valves are typically used because they can be integrated into the fluidic system with minimal requirements in terms of external components. In addition, the fluidic system can be fabricated in plastic for single-use operation, and the instrument can contain the external components required for valve actuation, which is a key attribute for IVD applications as the fluidic system can be configured as a disposable. Membrane valves typically employ a thin elastomeric material bonded to a rigid substrate containing the fluidic network and valve seats.

The common choice of elastomer for membrane valves in microfluidics is polydimethylsiloxane (PDMS). These so called “Quake valves” are fabricated using multilayer soft lithography.^26,27^ Numerous groups have used PDMS as an elastomer for pneumatically actuated valves with either glass or thermoplastic as the fluidic substrate.^21,28–31^ Although PDMS is known to bond strongly to glass following O_2_ plasma surface activation, PDMS unfortunately undergoes rapid hydrophobic recovery reducing its shelf life.^32^

As an alternative to glass, thermoplastics can be used for production of microfluidic devices or systems using various production modes, such as injection molding or extrusion. But for applications requiring valving, bonding of PDMS membranes to thermoplastics typically requires chemical modification^33,34^ to alter the surface chemistry to facilitate strong bonding.^35^ For example, valves composed of PDMS membranes bonded to COC substrates using 3-(trimethoxysilyl)propyl methacrylate modifier withstood pressures to 689 kPa.^34^

PDMS also has several limitations for its use in fluidic systems including non-specific adsorption of small molecules^36^ and limited compatibility with organic solvents resulting in PDMS swelling and dissolution of low molecular weight oligomers that can affect the integrity of the directed application.^37^ Therefore, alternative polymers have been proposed as valving membranes such as polycarbonate (PC),^13^ polyurethane,^38^ polypropylene,^39^ fluorinated ethylene propylene Teflon,^40,41^ fluoroelastomer,^42^ polystyrene,^43^ polyethylene glycol diacrylate,^44^ and polymethyl methacrylate (PMMA).^45^ A list of these materials is presented in Table 1. Two major conclusions can be reached based on information compiled in Table 1. First, these elastomer materials have favorable mechanical properties including reasonably low Young's modulus, meaning little pressure is required to actuate the membrane, and diverse values in terms of the elongation at break, which is the degree to which the material can be stretched before fracture. Second, complex fabrication and bonding techniques are required for microvalves to withstand high pressures, which does not lend itself well for applications requiring high-volume flow rates and/or channels with relatively small cross-sectional areas.

**Table 1:** Compilation of polymers proposed for membrane valves, the substrate material, the assembly method, and the highest pressure the valve was tested against without failure

Polymer	Properties		Thickness (μm)	Substrate	Assembly	Highest pressure reported (kPa)	Ref.
	Young's modulus (MPa)	Elongation at break (%)					
PDMS^a^	0.75	120	0.3–30	PDMS or glass	Multilayer soft lithography	200	26, 27, 48
PDMS^b^	1.2	325	254	Glass	Surface activation (UV/O_3_), thermal fusion bonding	75	28
PDMS^b^	1.2	325	254	PMMA	Surface activation (UV/O_3_)	60	21
PDMS^c^	1–2	140	1000	COP	Solvent (cyclohexane) and pressure bonded	24 000	49
Polycarbonate (PC)	2378	110	250	PC	Thermal fusion bonding	>690	13
Thermoplastic polyurethane (TPU)	26	600	25	PMMA	Solvent bonding (chloroform), thermal fusion bonding	>415	38
Polypropylene (PP)	1400	150–300	50	PMMA	Surface activation (UV/O_3_), thermal fusion bonding	>480	39
Fluorinated ethylene propylene (FEP) Teflon	480	300	25	Glass	Thermal fusion bonding	75	40
Fluorinated ethylene propylene (FEP) Teflon	480	300	12.5	PMMA	Adhesive bonding	500	41
Fluoroelastomer Viton®	N/A	>220	250	PMMA or COC	Solvent bonding (APTES/GPTES), thermal fusion bonding	400	42
						310	
Polystyrene (PS)	3000	30–40	25	PS	Solvent bonding (acetonitrile)	69	43
Polymerized polyethylene glycol diacrylate (poly-PEGDA)	130	5	40	Poly-PEGDA	Photopolymerization	>400	44, 50
Polymethyl methacrylate (PMMA)	2400	2.5–4	100	PMMA	Solvent bonding (chloroform)	100	45

a General Electric Silicones, RTV 615 (material properties reported in datasheet available from MG Chemicals and ref. 46).

b Bisco Silicones, HT-6135, HT-6240 (Young's modulus reported in ref. 47).

c Dow, Sylgard 184 (material properties reported in datasheets available from Dow Corning and ref. 46).

We present in this manuscript the use of elastomeric cyclic olefin copolymer (eCOC, TOPAS E-140) as a valving material that can be easily activated for attachment to a variety of plastic fluidic substrates without showing delamination when operated under high pressure, remains functional even after high number of actuation cycles, does not show rapid hydrophobic recovery, can be mass produced using either injection molding or extrusion, and is optically transparent. eCOC is a copolymer of polyethylene (PE) with a norbornene content <10%. This low norbornene and high PE content produces a material with mechanical properties that make it an ideal membrane material for valving. eCOC has an elongation at break >500%, which is higher than PDMS, and a low Young's modulus that results in low valve actuation pressures. Gleichweit *et al.* reported the use of eCOC as a membrane layer for doormat valves with COC.^51^ They reported two-component injection molding of the membrane material and thermo-compression bonding at 80 °C following UV/O_3_ activation and achieved a maximum bond strength of 186 J m^−2^. While they reported the valves to be leak-proof, no further characterization of the valves was performed. Additionally, COC was the only substrate reported for bonding to the eCOC membrane.

We also present here a novel “*in situ*” bonding method of eCOC to polycarbonate (PC) and polyethylene terephthalate glycol (PETG) that can be utilized to realize mass production of integrated fluidic systems with high process yield rates appropriate for IVD applications. To demonstrate the diverse range of operating conditions for eCOC as a membrane material for microvalves, we used both mechanical and pneumatic actuation with different thermoplastics.

Finally, we integrated eCOC valves into a modular system with a cell isolation module for the isolation of rare cells (*i.e.*, circulating leukemia cells, CLCs) from blood, and a module for immunophenotyping of cells (*i.e.*, imaging module) with the associated hardware for the detection of minimal residual disease (MRD) in acute lymphoblastic leukemia (ALL) patients. Monitoring of MRD is considered the most powerful predictor of outcome in acute leukemias, including B-type ALL (B-ALL). Our integrated system demonstrated the feasibility of MRD testing directly from blood, permitting frequent minimally invasive sampling of blood as opposed to a highly invasive bone marrow aspirate (BMA) to detect MRD.

## 2. Materials and methods

### 2.1. Reagents and materials

Cyclic olefin copolymer (COC) 6013 sheets were purchased from TOPAS Polyplastics (Farmington Hills, MI). COC E-140 (eCOC) pellets for extrusion were purchased from PolySource (Overland Park, KS). eCOC 100 μm thick films were obtained from TOPAS Polyplastics and Roehm (Parsippany-Troy Hills, NJ). OPTIX Acrylic sheets, polymethyl methacrylate (PMMA), and Lexan polycarbonate (PC) sheets were purchased from Polymershapes (Charlotte, NC). Zeonor-1060R, cyclic olefin polymer (COP), sheets were purchased from Knightsbridge Plastics Inc. (Fremont, CA). Polyethylene terephthalate glycol (PETG) sheets were purchased from McMaster-Carr (Elmhurst, IL). Polydimethylsiloxane (PDMS) film (HT6240, 250 μm) was purchased from Standard Rubber Products Co. (Elk Grovery Village, IL). Sylgard-184 (Dow Corning, Midland, MI) was used for PDMS casting in a 10 : 1 ratio. Microfluidic chips for cell isolation were purchased from Biofluidica, Inc. (San Diego, CA). PEEK tubing, IDEX part number 1569, was obtained from IDEX Health and Science (Oak Harbor, WA) and was connected to 3 mL Luer-Lok syringes (BD, Franklin Lakes, NJ) using Inner-Lock union capillary connectors (Polymicro Technologies, Phoenix, AZ) and barbed socket Luer lock fittings (McMaster-Carr). Epoxy used was Loctite® EA 9017 2-part epoxy adhesive (Henkel, Düsseldorf, Germany). Chemical reagents used included: isopropyl alcohol (IPA), 1× phosphate-buffer saline (PBS) (MidSci, Fenton, MO), food coloring dyes, 2-(4-morpholino)-ethane sulfonic acid (MES), saline sodium citrate (SSC), bovine serum albumin (BSA), Triton X-100, Tween®-20, ethanol, paraformaldehyde solution (Sigma-Aldrich, Saint Louis, MO), 1-ethyl-3-(3-dimethylaminopropyl)carbodiimide (EDC), *N*-hydroxysuccinimide (NHS) (Pierce, Rockford, IL), eptifibatide acetate (SML1042, Sigma-Aldrich), 1 M Tris pH 7.4 (KD Medical, Inc, Columbia, MD). The following antibodies were used in the studies: isolation antibody: human CD7 antibody (BioTechne, MAB7579, clone # 848438), human CD19 antibody (clone # 4G7-2E3R from R&D System); staining antibodies: anti-hcCD3 (Alexa Fluor® 750-conjugated, FAB100S) (R&D Systems, Minneapolis, MN), anti-TdT (Alexa Fluor® 647-conjugated, clone: E17-1519) (BD Biosciences, Franklin Lakes, NJ), DAPI (4′,6-diamidino-2-phenylindole) (Thermo Fisher, Waltham, MA), anti-hCD34 (Alexa Fluor® 750-conjugated, FAB72271S) (R&D Systems). The MOLT-3 (ATCC CRL-1552) cell line was obtained from the American Type Culture Collection (ATCC) and cultured in RPMI-1640 (ATCC, 30-2001) medium with 10% final concentration of fetal bovine serum (FBS, Performance, Gibco). Cells were maintained at an approximate concentration of 5 × 10^5^–2.5 × 10^6^ cells per mL and were counted with using an automated cell counter (Cellometer Auto T4, Nexcelom, Lawrence, MA).

### 2.2. Elastomeric cyclic olefin copolymer extrusion and injection molding

eCOC was extruded into films using a Lab Tech Engineering Company Ltd cast film extrusion line (Pittsburg State University (PSU), Pittsburg, KS) equipped with a single screw extruder (Milacron SMV-150). eCOC pellets were processed based on manufacturer guidelines. eCOC films, ranging from 100–500 μm thick, were extruded by varying the extrusion conditions as seen in Table 2. eCOC was also injection molded into 3 mm plaques using an Arburg Allrounder 320C injection molding machine (PSU) using manufacturer guidelines. Extruded eCOC films of 150 and 350 μm thickness and injection molded eCOC plaques were compared to 100 μm films from a commercial supplier using surface characterization techniques discussed in the following sections.

**Table 2:** Parameters used to cast film extrude eCOC films of specific thicknesses

Extruder speed (rpm)	Roll speed (ft min^−1^)	Nozzle temp (°F/°C)	eCOC film thickness (μm)
10	9–10	490/254	100–120
10	8	490/254	150–160
15	7	480/249	230–250
20	<9	480/249	>350

### 2.3. Activation of the polymer surface

Before any surface activation or surface measurements, all substrate surfaces were cleaned using isopropyl alcohol and nanopure water (18 MΩ cm), then dried in a 60 °C oven. Substrate surfaces were activated with either O_2_ plasma or UV/O_3_ exposure. O_2_ plasma activation was performed using a Femto plasma cleaner (Diener Electronic, Ebhausen, Germany) at 50 W, 20 sccm O_2_ gas flow, for 1 min. UV/O_3_ activation was performed using the UVO-Cleaner model 18 (Jelight Co, Irvine, CA) at an irradiance of 20 mW cm^−2^ at 1 cm from the light source, for varying amounts of time.

### 2.4. Water contact angle measurements

To quantify the extent of surface activation by the O_2_ plasma and UV/O_3_ producing lamp, sessile water contact angles were measured using a VCA Optima Instrument (AST Products, Billerica, MA, USA). Two μL of nuclease-free water was deposited onto the surface of the substrate and the software was used to measure the resulting water contact angle. At least three measurements were taken at various positions on multiple substrates. To test for hydrophobic recovery of eCOC and PDMS, water contact angles were taken at various time points (0–72 h) after 1 min O_2_ plasma surface activation. To evaluate the effects of UV/O_3_ exposure time on eCOC compared to other COC grades, water contact angle measurements were taken after the substrates were exposed to UV/O_3_ for 5, 10, and 15 min. To evaluate eCOC's shelf stability and hydrophilicity over time, water contact angle measurements were made on eCOC samples kept for 1 h, 1 day, and 18 months following 10 min UV/O_3_. Water contact angle measurements were additionally performed on both sides of eCOC substrates subjected to 10 min UV/O_3_ activation. The side facing upwards toward the UV lamp is referred to as the “frontside”, and the side facing opposite the activating source is referred to as the “backside”. Water contact angle measurements were used to determine whether the “backside” was hydrophilic following 10 min of UV/O_3_ activation. This was performed on eCOC of three different thicknesses (100, 150, 350 μm). Water contact angle measurements were also performed on pristine and UV/O_3_ activated COC, COP, PC, PMMA, and PETG.

### 2.5. Attenuated Total Reflectance Fourier Transform Infrared Spectroscopy (ATR-FTIR) measurements

ATR-FTIR measurements were performed using the Shimadzu IRAffinity-1S (Kyoto, Japan) with a platinum ATR module attachment on both the pristine and UV/O_3_ activated plastics. Collected spectra were analyze using the Shimadzu software and its compound IR library to identify functional groups and observe changes in the surface chemistry due to the activation.

### 2.6. Ultraviolet-visible (UV-vis) spectroscopy

A UV-vis spectrometer (Shimadzu UV-1280, Kyoto, Japan) was used to measure the transmittance of light through eCOC. Varying thicknesses of eCOC (130, 150, 280, 360 μm) were tested for their transmittance between 200 and 800 nm.

### 2.7. Design, fabrication, and assembly of test valves

**Fig. 1:**
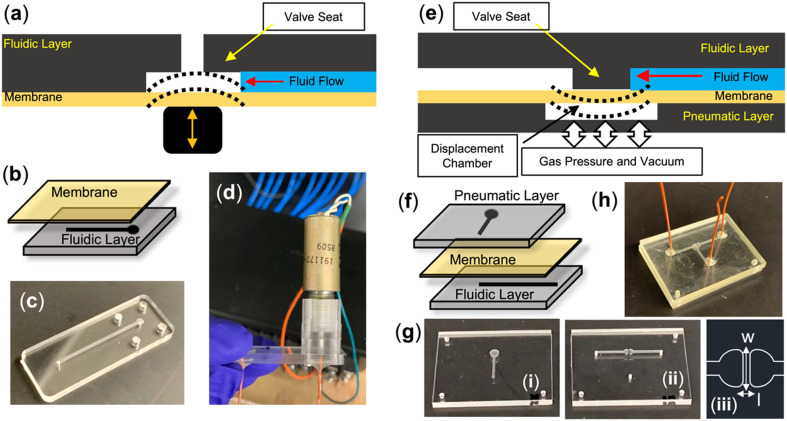
(a) Schematic of a mechanically actuated valve in which a solenoid pushes the valve membrane onto the valve seat to close the valve. (b) Visualization of a two-layer mechanical valve. Photos of (c) milled fluidic layer, and (d) fully assembled test valve with attached spacer and solenoid. (e) Schematic of a pneumatically actuated valve in which gas pressure and vacuum actuate the membrane onto the rigid fluidic layer to close and open the valve, respectively. (f) Visualization of a three-layer pneumatic valve. (g) Photos of milled (i) pneumatic layer, (ii) fluidic layer, (iii) schematic of the valve seat. (h) An assembled pneumatically actuated valve with PEEK connecting tubing lines.

To demonstrate the diverse range of operating conditions for the eCOC valves, two modes of actuation (*e.g.*, mechanical and pneumatic) were tested. Mechanically actuated valves consisted of two layers: a rigid thermoplastic fluidic layer and an elastomer layer as can be seen in Fig. 1a and b.

The fluidic layer included an inlet and outlet (diameter = 0.9 mm) joined by a microchannel (width = 0.9 mm, depth = 150 μm) with a single valve seat (diameter = 2 mm, depth = 150 μm) at the outlet. Individual test valve structures were milled into 3 mm thick sheets of five different thermoplastics using a desktop CNC milling machine (Nomad3, Carbide 3D, Torrance, CA). Milling parameters can be found in the ESI.† PMMA, PC, COP-1060R, COC-6013, and PETG were the thermoplastics chosen for these tests. An example of a milled substrate is shown in Fig. 1c. Ten min exposure to UV/O_3_ at 20 mW cm^−2^ was used to activate the surfaces and attach eCOC to the thermoplastic substrates.

A single stroke-push solenoid (Ledex/Johnson Electric SOL-102, Jameco part no. 195203-234) was attached onto the valve test structure using a CNC milled solenoid mount (Fig. 1d) and four screws. The mount had a fitting that matched the solenoid's 3/8′′ – 32 UNEF thread and allowed the solenoid's plunger to be aligned with the center of the valve seat and outlet so that when energized, the plunger pushed down on the eCOC and effectively closed the valve. PEEK tubing (1/32′′ OD × 0.02′′ ID) was epoxied to the inlet and outlet holes milled to have a matching diameter for tight fitting of the tubing. A fully assembled mechanically actuated test valve is shown in Fig. 1d.

Pneumatically actuated valves consisted of three layers: a rigid fluidic layer, a rigid pneumatic control layer, and an elastomer layer as shown in Fig. 1e and f.^21^ The microfluidic channel was 800 μm × 200 μm × 22.7 mm (*w* × *h* × *l*). The valve seat was 2 mm × 200 μm × 400 μm (*w* × *h* × *l*) as reflected in Fig. 1g. The pneumatic control layer contained a channel with an outlet for connection to a vacuum or compressed gas source that was used to actuate the elastomer layer. Milled pneumatic and fluidic layers are shown in Fig. 1g and an assembled valve in Fig. 1h.

For the pneumatically actuated test structures, the assembly was carried out as previously published.^14,21^ The fluidic layer and eCOC elastomer were first exposed to UV/O_3_ for 10 min and then the activated surfaces were pressed together with the elastomer sealing the fluidic channels. Then, the combined eCOC/fluidic layer was exposed to UV/O_3_ light along with the pneumatic layer for another 10 min. All three layers were then pressed together after aligning the pneumatic control displacement chamber to the valve seat in the fluidic layer. A PHI Press (TS-21-H-C (4A)-5, City of Industry, CA) was used with 750 lbf applied for 4.5 min followed by application of 1250 lbf for an additional 4.5 min. PEEK tubing (1/32′′ OD × 0.02′′ ID) was then epoxied to the inlet, outlet, and pneumatic control access channel network.

In both valving mechanisms, the elastomer layer sealed the fluidic network and was actuated by displacing the membrane onto the valve seat within the fluidic substrate to control fluid flow. eCOC (100 or 150 μm thickness) was cut from an extruded roll to the same footprint as the test valves (14.5 mm × 40 mm for mechanical and 40 mm × 30 mm for pneumatic) before bonding and assembly.

### 2.8. Burst pressure measurements

To quantify the bond strength between the thermoplastic microfluidic layer and eCOC membrane, burst pressure tests were performed.^35,52–54^ A syringe pump (New Era Pump System Inc. NE 1200, Farmingdale, NY) and a 100 psi (∼689 kPa) pressure sensor (OMEGA PX26 Series, PX26-100GV) were connected to the inlet line to monitor the pressure buildup within the fluidic network. The inlet line and microfluidic test chip were filled with 1× PBS and the solenoid was energized thus closing the valve. The pressure was allowed to build within the fluidic network using a volume flow rate of 20 μL min^−1^. At the burst pressure, the fluid broke through the bond between the thermoplastic fluidic substrate and elastomer membrane cover causing delamination and a rapid decrease in pressure as indicated by the pressure sensor. Burst pressure tests were performed for two-layer devices made from each of the five thermoplastics bonded to eCOC (COC/eCOC, COP/eCOC, PC/eCOC, PMMA/eCOC, and PETG/eCOC).

Burst pressure measurements were also performed with a third layer added to the fluidic device. This rigid cover layer was made from PMMA with various thicknesses (250, 500, 800, 3000 μm) and was assembled to the top of the two-layer PMMA/eCOC device.

### 2.9. Mechanically actuated eCOC valve characterization

PETG/eCOC two-layer devices were utilized to demonstrate the ability to actuate the eCOC membrane valves mechanically using a solenoid. PETG/eCOC test valves were assembled using 10 min UV/O_3_ activation and stroke-push solenoids were attached using the method described in section 2.7. The solenoid was connected to a power switch that allowed for actuation of the valve. Using the same burst pressure setup as described in section 2.8, pressure was allowed to build within the test valve up to 100 kPa, the syringe pump was turned off, and the valve was held closed for 20 min. To test the number of actuations the valve could withstand, this experiment was repeated following 50 actuations.

### 2.10. Pneumatically actuated eCOC valve characterization

To demonstrate eCOC's ability to act as a membrane valve with pneumatic actuation, pneumatic test valve seats were milled into PMMA. This pneumatic layer was then bonded to the eCOC/PMMA fluidic layer as described in section 2.7. The three-layer PMMA/eCOC/PMMA chip was connected to a previously reported pneumatic control system.^14^ This control system consisted of a series of software controlled 3/2 way solenoid valves (M1533724VDC, Humphrey Products, Kalamazoo, MI) that were connected to a gas tank and a vacuum source. The applied gas pressure ranged from 5 to 110 kPa and the applied vacuum was −50 kPa.^14^

Each test valve underwent a series of trials to characterize the thermoplastic/eCOC valve's tolerance to flow rate, fluidic pressure buildup, closing pressures, and failure modes. A syringe pump was connected to the inlet of the device to supply fluid to the system and allow for pressure build up when the valve was closed. To monitor the pressure, the same pressure sensor as mentioned previously was added in series between the syringe pump and valve. To observe the valve seat, a Dino-Lite Microscope was placed directly above the test valve structure. Various closing pressures ranging from 5 to 110 kPa were applied by controlling the gas pressure. For each test valve, the full range of closing pressures were applied and using the microscope and pressure sensor, leakage pressures were quantified. The number of actuations cycles the valve could tolerate was also noted. When delamination occurred, the valve became unusable, and the conditions that caused delamination were noted.

### 2.11. Integrated system for selecting and immunophenotyping circulating leukemia cells (CLCs)

To demonstrate eCOC's ability to work as a valve membrane within an integrated system, we used SMART-Chip^14^ for isolation of CD7(+) model cell line and CD19(+) clinical circulating leukemia cells (CLCs) isolated from the blood of a B-ALL pediatric patient. Immunophenotyping of the isolated cells was also performed on the integrated system using an imaging module. The impedance counter that was used in the SMART-Chip for epithelial circulating tumor cells (CTCs) counting was not used in this application. The reason being the fact that unlike CTCs that are rare in blood (typically cells with epithelial markers do not circulate in blood), CLCs and normal white blood cells express the same surface antigens (*i.e.*, CD7 or CD19) used for affinity isolation. Because affinity isolated cells represent both populations, aberrant cells must be identified based on immunophenotyping (*i.e.*, anti-TdT antibody staining of TdT) within the imaging module. Therefore, the cell counting module was deemed not useful and was replaced with a jumper module. While the jumper module served only to allow transfer of fluid from the cell selection module to the imaging module, it allowed the use of the same motherboard as used previously without requiring a new design increasing its operational portfolio.^14^ In addition, because the jumper module is made from a plastic and can be injection molded, it can be mass produced at a cost <$2, which only contributes minimally to the total cost of the SMART-Chip.

eCOC also replaced the previously used PDMS as the membrane layer within the motherboard for pneumatic actuation. The motherboard consisted of a fluidic layer and pneumatic control layer both fabricated out of PMMA. The fluidic layer contained the microchannels, 11 valve seats, and the conical ports (top diameter = 1.7 mm, bottom diameter = 1.4 mm, depth = 2.2 mm) to attach the modules to the motherboard. The pneumatic control layer contained the displacement chambers.^14^ After direct milling of the required structures in the PMMA layers, the substrates were cleaned, and *ex situ* bonded with the same procedure described in section 2.7. A custom made jig was used to align the fluidic and pneumatic layers.

The preparation of the cell selection and imaging module have been previously published^14,55,56^ and can be found in more detail in the ESI.† After preparation, the cell selection module, jumper module, and imaging module were connected to the motherboard through the conical ports using semirigid Tefzel tubing.^13^ PEEK tubing was then connected to the motherboard as fluidic and pneumatic inputs. PEEK tubing (OD 1/16′′) from the motherboard was connected to a solenoid control system.^14^

### 2.12. Clinical samples

Healthy donor blood samples were obtained from the Biospecimen Repository Core Facility at the University of Kansas Medical Center (KUMC) under an approved IRB. Pediatric patients (1 to 19 years of age) treated at Children's Mercy Hospital in Kansas City, MO and were diagnosed with precursor B-cell ALL were also evaluated. Blood samples were collected according to an approved Children's Mercy Hospital Institutional Review Board procedure (STUDY00001508). Written informed consent was obtained from the patient before enrollment. For both healthy donors and B-ALL patients, peripheral blood samples (5 mL) were drawn by venipuncture into Vacuette® containing EDTA (Greiner) tubes.

### 2.13. Model cell line and clinical sample testing using the modular system

MOLT-3 cells were taken from culture and centrifuged at 300 × *g* for 7 min, washed with PBS three times, and resuspended in 500 μL PBS. B-ALL clinical samples were acquired from Children's Mercy Hospital in Kansas City, Missouri, as noted above. Both MOLT-3 cell suspensions and clinical samples were run on the SMART-Chip including through a COP sinusoidal chip functionalized (PC-linker) with either anti-CD7 or anti-CD19 mAb (see ESI†). The 11 valves were used to connect the reagents and control the flow of fluid through each module that were interconnected to the fluidic motherboard. By opening valve 1, 1 mL of 0.5% BSA/PBS (4 mm s^−1^ linear velocity) was pumped through the cell isolation module using a syringe pump (Harvard Apparatus, Holliston, MA) and was directed to waste by closing valves 2, 3, and 5 and opening valve 4. While keeping valve 4 open and valve 5 closed, the following steps were performed sequentially to direct fluid throughout the cell isolation chip and to waste: valve 1 was closed, and valve 2 was opened to run either 500 μL of MOLT-3 cell suspension or 2–4 mL of blood (2 mm s^−1^ linear velocity); valve 2 was closed, and valve 1 was reopened to wash with 1 mL 0.5% BSA/PBS (4 mm s^−1^ linear velocity); valve 1 was closed and valve 3 was opened to wash with 500 μL PBS (4 mm s^−1^ linear velocity). After the PBS wash, the cell isolation module was exposed to 405 nm wavelength light for 3 minutes.^57^ Keeping valves 1 and 2 closed, valve 3 was opened to wash with 1 mL PBS at 10 μL min^−1^. To direct released cells to the imaging module during this step, valve 4 to waste was closed and valves 5 and 6 were opened thus directing the fluid from the cell selection module to imaging module.

After cells were introduced to the imaging module following release from the cell selection module, valve 6 was kept closed to direct fluid through the imaging module and towards waste and valves 7–11 connecting the reagents were all kept closed unless specified. Cells were washed briefly (20 μL at 10 μL min^−1^) with PBS by opening valve 11. A flow rate of 10 μL min^−1^ was used in every step with the imaging chip, as previously published.^58^ Following the wash, cells were fixed with 4% PFA in PBS for 10 min at room temperature by opening valve 7 and permeabilized with 0.1% Triton X-100 in water for 15 min by opening valve 8. The intracellular stains (*i.e.*, TdT and cCD3 or CD34 at 5.0 μg mL^−1^ concentration in 0.2% BSA/0.2% Tween-20 and PBS) were introduced into imaging module by opening valve 9. Staining was performed for 40 min at room temperature. Cells were washed with 50 μL of 0.05% Tween-20 in PBS by reopening valve 9 with the different reagent attached. Finally, by opening valve 10 the cells were stained with DAPI in PBS for 5 min, and the device was rinsed with PBS by opening valve 11 and imaged using a fluorescent microscope.

The cells were imaged using a Zeiss 200 M Axiovert inverted epifluorescence microscope (Carl Zeiss, White Plains, NY) equipped with an XBO-75 lamp and a Cascade 1K CC camera (Roper Scientific, Tucson, AZ). Images were collected with the following acquisition times: DAPI (20 ms), AF-647 (Cy5 filter, 3000 ms), AF-750 (Cy7 filter, 5000 ms). Images were processed using FIJI by ImageJ software (National Institute of Health, Bethesda, MD).

## 3. Results and discussion

### 3.1. eCOC chemical, thermal, and mechanical properties

eCOC is a copolymer consisting of ethylene and norbornene monomers with different COC grades defined by the ratio of the ethylene to norbornene content. The unique elastomeric nature of eCOC arises from the low percentage of norbornene (<10%) with respect to the ethylene content.^59,60^ The monomer percentage in the COC grade affects the thermal and mechanical properties of the copolymer, such as the glass transition temperature (*T*_g_), Young's modulus, and elongation at break. As seen in Table 3 for different COC grades, as the norbornene content increases, the *T*_g_ and Young's modulus increase while the elongation at break decreases. The Young's modulus and elongation at break are two important mechanical properties of any valving membrane. In terms of valves, the Young's modulus determines the amount of force required by the actuation process (*i.e.*, pneumatic or mechanical) to displace the membrane from its equilibrium position to a position to actuate the valve. This relationship is demonstrated in eqn (1), where *P* is the applied pressure, *r* is the valve radius, *E* is Young's modulus, *h* is the membrane thickness, *v* is Poisson's ratio, and *y* is the deflection of the membrane:^44,61^1
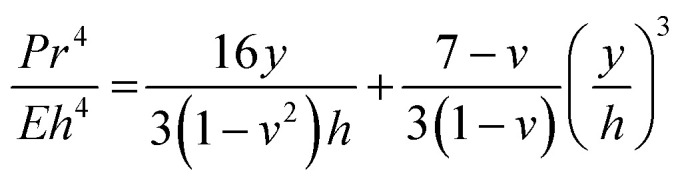


**Table 3:** Thermal and mechanical properties of materials used in the study^62,63^

COC grade	Norbornene (mol%)	*T*_g_ (°C)	Young's modulus (MPa)	Elongation at break (%)
6017	60	170	3000	2.4
6013	50	130	2900	2.6
8007	35	75	2600	4.5
**E-140**	**<10**	**<15**	**50**	**>500**
PE	0	<0	200–300	400

In contrast, the elongation at break is the percentage change of the membrane material length under tension before material failure (*i.e.*, exceeds its elastic and plastic deformation regimes and fractures). Comparing eCOC (E-140) in Table 1 to PDMS and other polymers, eCOC has an elongation at break >500% whereas PDMS and other elastomers have values ≤350%. Additionally, while the Young's modulus of eCOC is nearly 10-fold larger than PDMS, it is 2–60× lower than other plastics listed in Table 1. The consequences of these elastomer material properties for microfluidic valving are significant in terms of the pressure needed to actuate the valve and the valve architecture, in particular the depth of the valve seat (*i.e.*, larger elongation at breaks means a deeper valve seat can be used). For example, we reported an integrated modular system for genotyping^13^ and this system used a PC membrane valve, which has an elongation at break of 100%. Therefore, a small distance between the valve seat and membrane (<100 μm) was required to stay within the elastic regime of PC, which required tight valve assembly conditions for successful fabrication and operation. Also, because of the shallow valve seat, valve failure was frequently observed due to “sticking” of the membrane to the PC valve seat especially during device assembly, which utilized thermal fusion bonding. For the valves reported herein, we could use distances between the valving membrane and the valve seat >150 μm resulting in a process yield rate of 100% both in terms of valve assembly and valve operation.

For its thermal properties, eCOC has a *T*_g_ ranging from −6 to +15 °C (ref. 59 and 64) in comparison to the other COC grades, which have *T*_g_'s >75 °C (Table 3). Thermal fusion bonding, typically used for thermoplastic microfluidic assembly, occurs at or slightly below the *T*_g_ of the substrate and/or cover plate.^65–67^ With the use of eCOC, it can be bonded to the substrate at or near room temperature, which would result in minimal amounts of microstructure or even nanostructure deformation during assembly.^68^

#### 3.1.1. Surface chemistry of eCOC

The surface properties of a valving membrane in microfluidics are important in terms of its wettability, propensity to mitigate non-specific adsorption, and mechanical stability. For example, PDMS can be made more wettable using an O_2_ plasma (change in water contact angle from 105° ± 10°, to 0° immediately after activation), but shows rapid hydrophobic recovery (water contact angle recovers to 96° ± 5.4° <72 h). In comparison, photo-oxidation of eCOC can use UV/O_3_ or O_2_ plasma and its surface remains stable for ≫72 h (water contact angle of 73° ± 2.5° after O_2_ plasma activation) as shown in Fig. S1.† Hydrophobic recovery in PDMS is widely reported to be due to its low molecular weight species migrating back into the bulk.^32^

Surface activation using UV/O_3_ or O_2_ plasma has been shown to increase the surface hydrophilicity of many thermoplastics.^63,66,69–73^ The water contact angle for COC, COP, PC, PMMA, and PETG can be found in Fig. S2.† Each plastic exhibited a decrease in its water contact angle following surface activation due to photo-oxidation reactions that create surface-confined carboxylic acids and other oxygen-containing groups.^63^ Photo-oxidation can result in polymer chain-scissioning creating surface-level hydrophilicity, polar groups, and smaller polymer chains.^63,69^ These changes can assist in thermal fusion bonding of plastics by lowering the local *T*_g_ and mobility of the polymer chains to allow facile bonding. Additionally, COC hydrophobic recovery following activation/oxidation is nearly inconsequential.^69,74^

**Fig. 2:**
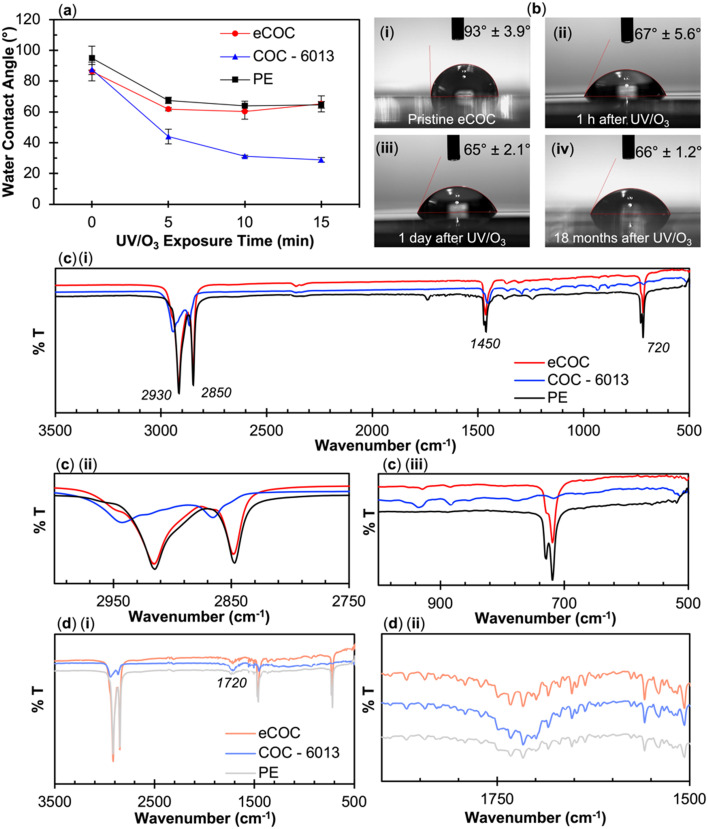
(a) Water contact angle measurements of eCOC, COC-6013, and PE showing the impact of the norbornene content on surface activation. (b) Water contact angle pictures of eCOC; (i) before UV/O_3_ exposure and various time periods after UV/O_3_ activation; (ii) 1 h; (iii) 1 day; and (iv) 18 months. (c) (i) ATR-FTIR results for pristine eCOC, COC-6013, and PE. Zoomed in regions of interest are shown between; (ii) 2750–3000 cm^−1^ for –CH stretching mode; and (iii) and 500–1000 cm^−1^ for C–CH_2_ rocking. (d) (i) ATR-FTIR results for UV/O_3_ activated eCOC, COC-6013, and PE with; (ii) zoomed in region around 1700 cm^−1^, carbonyl region.

Water contact angle measurements and ATR-FTIR measurements were performed on different COC grades before and after activation using UV/O_3_ treatment with comparisons made to eCOC. Our group has previously characterized the surface chemistry of different COC grades following UV/O_3_ activation and reported high norbornene content in the COC results in lower water contact angles following UV/O_3_ exposure.^63^ Based on these observations, eCOC would be expected to have fewer oxygen-containing species following UV/O_3_ activation and thus less hydrophilic character. Indeed, the water contact angle of eCOC generated a higher water contact angle following activation compared to COC-6013 (see Fig. 2a). While the eCOC surface became hydrophilic following UV/O_3_ activation (change in water contact angle from 86° ± 6.0° to 60° ± 4.9°), the water contact angle did not change as significantly as it did for COC-6013 (change in water contact angle from 87° ± 3.2° to 30° ± 1.0°) following UV/O_3_ activation under the same conditions. The water contact angle for eCOC did not further decrease when longer UV/O_3_ treatment was implemented (see Fig. 2a and ESI†).

To evaluate whether eCOC was stable after UV/O_3_ activation, water contact angle measurements were collected at various time points after activation (1 h, 1 day, and 18 months), and the results are shown in Fig. 2b. Even after 18 months, the water contact angle for eCOC remained at 66° ± 1.2°, close to that immediately following activation.

ATR-FTIR was also performed on eCOC, PE, and COC-6013 as previous data from our laboratory has shown that ATR-FTIR can be used to search for functional groups appearing after UV/O_3_ activation of many plastics, such as the appearance of carbonyl groups formed due to photo-oxidation reactions.^63,69^Fig. 2c(i) compares the pristine surfaces of the three polymers. There are four infrared bands that are consistent with previous findings.^51,63,75^Fig. 2c(ii) highlights the first noticeable feature spanning the 2930–2850 cm^−1^ range, which are indicative of –CH stretching vibrations for all three plastics as well as a band at 1450 cm^−1^ due to a –CH bending mode. Fig. 2c(iii) shows a band at ∼720 cm^−1^ that appears in PE and eCOC but not in COC-6013. Peaks at 720–730 cm^−1^ represent a –CH_2_ rocking vibration.^76,77^ Split peaks at this wavenumber are also indicative of long chain alkanes.^78^Fig. 2d(i) shows the ATR-FTIR results following UV/O_3_ activation for 10 min for each plastic. A peak at ∼1700 cm^−1^ is representative of a carbonyl group that was seen in all three plastics as highlighted in Fig. 2d(ii). This is evidence of oxygen-containing species (ketones, aldehydes, hydroxyls, and carboxylic acids) formed due to surface activation.^63^

#### 3.1.2. eCOC fusion bonding to thermoplastic substrates

**Fig. 3:**
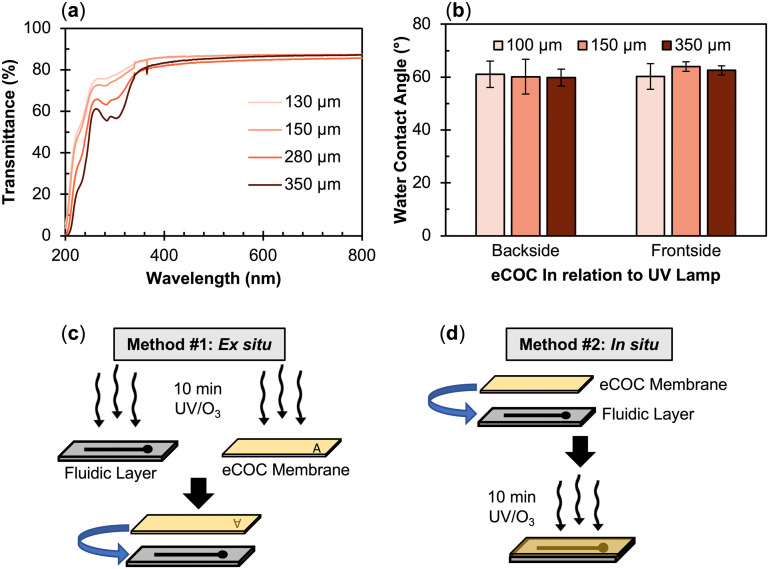
(a) UV-visible spectrum showing transmittance for UV/O_3_ activated, various thicknesses of eCOC (130, 150, 280, and 350 μm). (b) Water contact angle of various thicknesses of eCOC (100, 150, 350 μm) comparing the backside and frontside of the eCOC in relation to the UV lamp after 10 min UV/O_3_ exposure. (c) Method #1: *ex situ*, 10 min UV/O_3_ activation and manually pressing the activated surfaces together. (d) Method #2: *in situ*, using 10 min UV/O_3_ activation through the eCOC layer to bond the layers together.

Material characterization was performed on different thicknesses of extruded eCOC films. All eCOC films became hydrophilic following UV/O_3_ activation (between 60–70°) and showed formation of carbonyl containing functionalities as deduced from ATR-FTIR spectra (see Fig. S3†). Fig. 3a shows the UV-vis spectra for UV/O_3_ activated eCOC ranging in thicknesses between 130 μm and 350 μm. UV-vis spectra revealed that 130 μm thick eCOC transmitted ∼70% of UV light at 254 nm even after 10 min UV/O_3_ activation. The UV-vis spectra for pristine eCOC can be found in Fig. S4.† We did not observe a significant decrease in UV transmittance at 254 nm following UV/O_3_ activation (75% *vs.* 73% transmittance for pristine and post-UV/O_3_ activation, respectively) as is commonly seen in other thermoplastics.^69^ As expected, when the thickness of the eCOC increased, the transmittance at 254 nm decreased to 40% for a 350 μm thick film. Interestingly, when the water contact angle of the “backside” of the eCOC was tested, the water contact angle was 60° ± 5°, similar to values observed for the “frontside” of the eCOC, which was the side directly facing the UV/O_3_ source (average 62° ± 3.5°)_._ The data suggests that even with 60% of 254 nm light absorbed, the UV light had sufficient energy to activate the “backside” of the eCOC, as indicated by water contact angle measurements shown in Fig. 3b.

These results imply that it is possible to activate the material in proximal contact with the backside of eCOC. To test this hypothesis, we exposed different thermoplastics to 254 nm light with a 100 μm thick eCOC film placed over the plastic. The bonding conditions to different thermoplastics was also performed to demonstrate the broad utility of eCOC as a valving membrane for different substrates. Surprisingly, PC and PETG substrates positioned underneath the eCOC film strongly fusion bonded to eCOC when exposed to UV/O_3_ at room temperature. PMMA, COC, and COP, however, did not bond to eCOC using these conditions.

To confirm surface activation of plastics under the eCOC film, thermoplastics were exposed to UV light through a 100 μm thick eCOC film placed on top of a quartz slide (1 mm thick) with the plastic substrate placed beneath the quartz slide, which prohibited bonding of the eCOC film to the underlying plastic to allow for surface interrogation of the plastic. The UV/O_3_ source emits at 185 nm and 254 nm; 185 nm light helps to generate ozone while 254 nm light destroys the ozone, thus irradiating the eCOC and the bottom plastic with atomic oxygen and generating a photo-initiated oxidation reaction on the surface. The eCOC and quartz sitting on top of the plastic surface also prevented ozone from physically reaching the thermoplastic's surface and because eCOC absorbs at 185 nm, it acts as a barrier to ozone formation at the eCOC/quartz/plastic interfaces. Fig. S2† shows the experimental set up and the water contact angle results for the various thermoplastics irradiated under these conditions. After 10 min exposure, with and without eCOC/quartz situated on top of the PETG surface, the water contact angle of PETG decreased from ∼80° to ∼35°. As can be seen in Fig. S2,† all other thermoplastics saw a decrease in their water contact angle through the eCOC/quartz but none as dramatic as PETG.

Discerning the functional group identity of PETG activation after UV/O_3_ exposure was difficult using ATR-FTIR (Fig. S5†) because this polymer's structure is already composed of a variety of functional groups such as carboxylic acids, methylene groups, ester groups, benzene, and cyclohexane rings.^79^ PETG activation with UV/O_3_ is similar mechanistically to PC,^69^ which occurs *via* photo-Fries chemistry driven by high energy UV light <300 nm.^79–82^ The mechanism of PETG activation by UV proposed by Grossetête *et al.*^80^ indicated that the aromatic chromophores absorb the incident radiation within the first 5 μm of the PETG surface creating photoproducts consisting of highly reactive radicals accompanied by polymer chain scissioning reactions (see Fig. S6†).

Based on the results presented above, we propose two different eCOC valve membrane bonding methods (Fig. 3c and d). In the first method, called “*ex situ*”, the eCOC elastomer layer and substrate surface are exposed to UV/O_3_ for 10 min and the activated surfaces are pressed together under controlled pressure. This procedure was the necessary bonding method for PMMA to eCOC as an example. In the second method, called “*in situ*”, the eCOC elastomer layer and substrate are brought into conformal contact followed by activation with UV/O_3_ for 10 min. This bonding method could be used for PC or PETG substrate bonding to eCOC. The *in situ* method is made possible due to eCOC's high UV transmittance (254 nm), which activates both eCOC and the substrate to create a fusion bond between eCOC and the thermoplastic substrate. Neither of these methods requires added adhesive/solvent, elevated heat, or regulated high applied pressure to induce bonding. Thus, the *in situ* method could provide a better route for mass production as this method has a simpler process flow as well as minimal deformation of the underlying microstructures situated on the fluidic substrate because the temperature (*i.e.*, room temperature) is below the glass transition temperature of the substrate. Thus, a higher process yield rate would result.

### 3.2. Evaluation of eCOC/thermoplastic valve's burst pressure

**Fig. 4:**
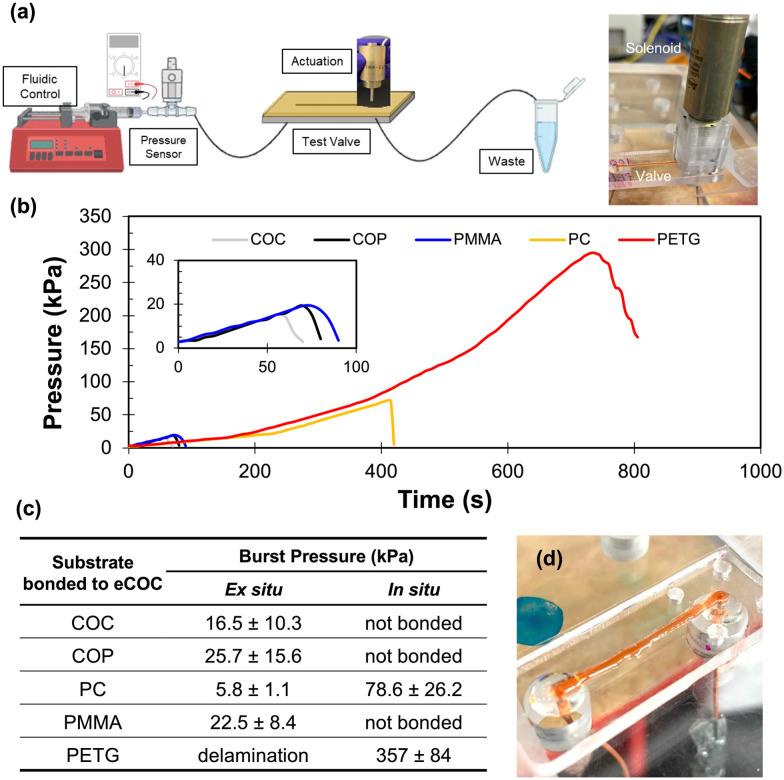
(a) Set up for burst pressure testing using valves assembled using either assembly method depicted in Fig. 3. Created with Biorendor. (b) Burst pressure test results showing increase in pressure over time until each valve assembly hits its burst pressure and there is a rapid decrease in pressure representative of device failure and delamination. The inset shows an expanded view of this data with the same units. (c) Burst pressure results for eCOC bonded to COC, COP, PC, PMMA, and PETG based on assembly method (at least *n* = 3). (d) Photo of eCOC/PETG test valve using *in situ* bonding holding at 340 kPa.

To determine the fusion bonding strength of the eCOC elastomer to a thermoplastic substrate, burst pressure measurements were performed using mechanically actuated test valves. These measurements were intended to mimic the high fluidic pressures typically encountered in microfluidic systems.^35^ eCOC membranes were assembled to five different thermoplastic substrates (COC-6013, COP, PC, PMMA, and PETG) using two different assembly conditions (Fig. 3c and d). The burst pressure testing system is depicted in Fig. 4a. A 100 psi pressure sensor was connected in series with a syringe pump and the closed valve to monitor the pressure buildup within the fluidic network. At the burst pressure, a rapid decrease in pressure occurs (Fig. 4b). Because the eCOC elastomer is flexible, when the solenoid plunger pushes the eCOC onto the valve seat it creates a tight seal and does not allow fluid to leak through the closed valve. For the valve to fail at the burst pressure, fluidic pressure would either need to push the solenoid plunger backwards off the valve seat (unlikely) or result in delamination of the eCOC membrane bonded to the thermoplastic substrate (likely). The burst pressure can therefore be used as a quantitative metric to determine operational parameters of the valve.

All burst pressure data were collected for at least 3 different valves as delineated in the table of Fig. 4. *Ex situ* bonding (*i.e.*, activation with UV/O_3_ before bonding) resulted in <25 kPa burst pressures for all thermoplastic/eCOC combinations. PETG/eCOC test valves failed with the introduction of liquid, PC/eCOC was not able to withstand more than 6 kPa, while COC, COP, and PMMA were able to withstand pressures from 15 to 25 kPa. It was observed that at the burst pressure, leakage resulted from delamination of eCOC from the substrates in all cases as expected.

For the *in situ* bonding process, this resulted in bonding only between PC/eCOC and PETG/eCOC. COC, COP, and PMMA did not bond at all to eCOC using this method. PETG/eCOC test valves withstood fluidic pressures of 357 ± 84 kPa (*n* = 6) without delamination. This was 4× higher than the PC/eCOC valve assembly, which could withstand fluidic pressures of 78.6 ± 26.2 kPa, which was still >14× higher than burst pressures for the *ex situ* valve assemblies with PMMA, COC, and COP substrates.

PETG and PC bonding to eCOC using the *in situ* process was hypothesized to result from formation of highly reactive products following photo-oxidation reactions that occurred in PC and PETG after UV/O_3_ exposure, which includes formation of a number of different radicals (see Fig. S6†).^79,80,83^ These photo-products can induce reactions with eCOC when both are in proximity (*i.e.*, active radicals on both surfaces). Aromatics within the backbones of PETG and PC are part of the photo-oxidation mechanism; COC, COP, and PMMA lack aromatics and thus do not undergo photo-Fries type reactions. However, Nakade *et al.* showed the formation of radicals within COC following photo-induced activation.^84^ Therefore, the high numbers of radicals formed in PETG and PC can induce cross-linking with reactive polymer species formed within eCOC^84,85^ and thus, form strong bonds; in some cases resulting in polymer chain cross-linking. As a reference, higher norbornene containing COC-8007 (35% norbornene) did not bond to PETG. This may be due to COC-8007's *T*_g_ being above room temperature, and its chains are thus less mobile preventing cross-linking reactions to PC and PETG during *in situ* bonding. In addition to radical formation, COOH-terminated material form transient bonds, which allow stress release by a bond-partner exchange mechanism creating covalent bonds.^86^

PETG/eCOC samples bonded using the *in situ* method were observed to stay strongly bonded for >18 months. A PETG/eCOC valve tested >6 months after bonding was still able to successfully operate at pressures >340 kPa. This indicates that the *in situ* bonding method between PETG or PC and eCOC would provide long shelf lives.

**Fig. 5:**
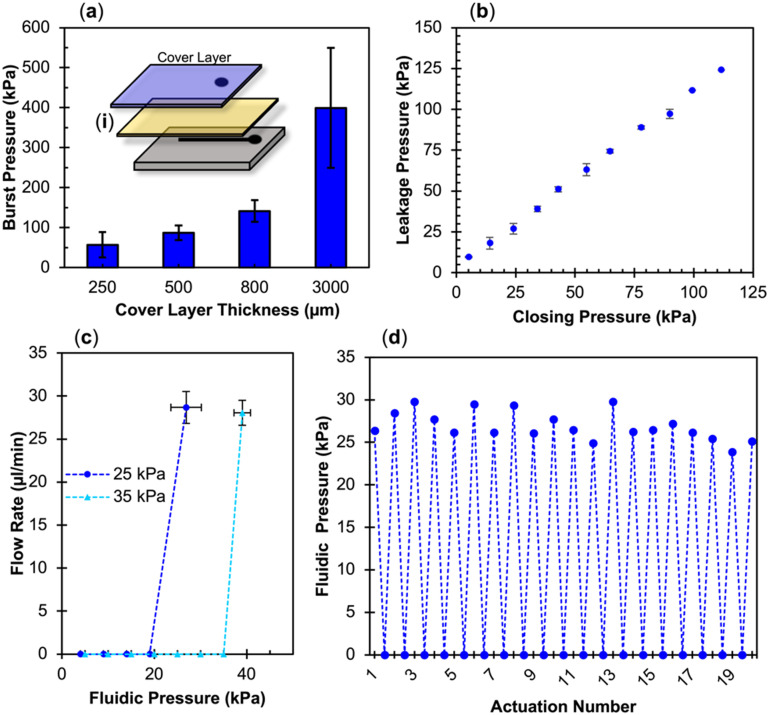
(a) Burst pressure of *ex situ* bonded three-layer valves based on thicknesses of the added cover layer (*n* = 3) with an inlay (i) schematic of mechanical actuation valve with added cover layer. (b–d) Pneumatic actuation results using eCOC as the membrane layer: (b) closing pressure *vs.* leakage pressure for PMMA to eCOC (*n* = 3). (c) Fluidic pressure needed to cause leakage for two different closing pressures (25 and 35 kPa) for PMMA/eCOC/PMMA valves (*n* = 3). (d) Leakage pressure for 20 actuations for PMMA/eCOC/PMMA test valve.

The adhesion strength of PC and PETG with eCOC exceeded eCOC's elasticity under high fluidic pressures as shown in Fig. 4d. In this case, eCOC started to deform and bulge under high fluidic pressure. While this bonding is impressive, deformation of the eCOC under high fluidic pressures can have unwelcomed consequences such as inconsistent liquid flow parameters (*i.e.*, linear velocity) due to an unintended change in the fluidic channel's cross-sectional area. eCOC extruded films thicker than 170 μm were not able to reliably bond to PETG using *in situ* bonding, which may be due to decreases in UV transmittance with increasing eCOC thickness. We therefore added a rigid cover layer on top of eCOC that would restrict its deformation under high fluidic pressures. We tested the burst pressure with the setup shown in Fig. 4a but in this case, with the addition of a bonded rigid cover layer as shown in Fig. 5a(i); this created a three-layer valve in contrast to the two-layer valves presented earlier. PMMA was selected as the rigid cover layer and substrate valve material due to its high Young's modulus, high *T*_g_, and simplicity in machining (see Table 1).

The use of a hard cover layer in the three-layer valve system (Fig. 5a) resulted in higher burst pressures as compared to the two-layer valve depicted in Fig. 4c. In particular, the three-layer configuration (PMMA/eCOC/PMMA) resulted in a burst pressure of 400 ± 150 kPa. Using the two-layer system, increasing fluidic pressure within the channel resulted in deforming the highly elastic eCOC layer and eventually delamination of the membrane from the thermoplastic substrate resulting in valve failure. The addition of a rigid layer of PMMA that is much stiffer than eCOC (3378 MPa and 50 MPa, respectively; Table 1), provided the ability to tolerate higher fluidic pressures compared to the two-layer valve configuration. Young's modulus is calculated for deformation under tension or compression; however, the cover layer can be considered fixed and when the pressure begins building within the fluidic channel of the valve, pressure pushes upwards on the membrane causing bending of the material. While eCOC is highly flexible, especially at 100 μm thicknesses, one needs to consider the flexural modulus of the PMMA cover layer. In particular, the PMMA thickness contributes to its stiffness, as the stiffness is proportional to the cube of the thickness as seen in eqn (2) for flexural modulus (*E*_flex_) where *h* is the height or thickness of the material, *L* is the length, *F* is the force applied, *w* is the width, and *d* is the displacement caused by *F*;^87^2
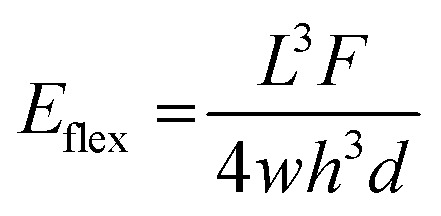
This was evident in the increased burst pressure as the thickness of the PMMA cover layer increased. For example, keeping all other variables constant, increasing the thickness from 500 μm to 800 μm increases the flexural modulus by 4-fold while increasing the thickness from 800 μm to 3000 μm increases the flexural modulus by 50-fold. It should be noted that the burst pressures in Fig. 5a do not scale equally to increases in the flexural modulus.

### 3.3. eCOC mechanical actuation

To demonstrate eCOC's ability to work as a valving membrane, *in situ* bonded PETG/eCOC mechanically actuated two-layer valves were tested using a test valve embedded within a fluidic network. The parameters that were evaluated included the ability to not leak at typical system operational pressures and the number of actuation cycles the valves could withstand before failure. These testing parameters were based on requirements needed as delineated from our previous reports on using integrated and modular microfluidic systems.^14^ Our goal was to achieve the following operational characteristics for our eCOC valve: (i) holding pressure of ∼50 kPa for at least 20 min; (ii) actuation numbers ≥50; and (iii) valve holding time >20 min with no “sticking” to the valve seat. No sign of delamination during operation of the test valve was observed for a total operating time of 40 min. In addition, after holding the valve closed for 20 min there was no evidence of eCOC sticking to the PETG valve seat, which would be evident in pressure build up even when the solenoid had been powered off and returned to the “open state”. The three-layer test valves containing an 800 μm PMMA cover *ex situ* bonded to a PMMA/eCOC assembly held 48 kPa for ∼30 min, could be actuated 50 times, and could be held closed for >30 min without sticking to the valve seat.

The reason for this high number of actuations without eCOC valve failure is due to its high elongation at break (>500%). eCOC does not undergo permanent plastic deformation that could cause collapse of the membrane onto the valve seat following actuation. This high elongation at break also allowed for large distances (>150 μm) that could be tolerated between the valving membrane and valve seat, thus lessening the likelihood of failure due to sticking or collapsing of the membrane onto the valve seat. Also, this relaxes tight tolerances required for the fabrication, increasing the process yield rate of device fabrication.

### 3.4. eCOC pneumatic valve actuation

For pneumatically actuated valves, as the fluidic pressure builds at a closed valve, operational failure can occur in two ways. First, if the bond strength between the thermoplastic and the elastomer membrane is weak, the fluid can begin to flow between layers causing delamination. If the bond strength between the elastomer and thermoplastic is strong, then the fluid begins to push on the elastomer at the valve seat. The second scenario of failure would occur when the fluid pressure reaches or exceeds the gas pressure closing the valve allowing uncontrollable liquid flow. This is called leakage pressure. As shown in the mechanical actuation results, three-layer PMMA/eCOC/PMMA valves were able to withstand burst pressures up to 400 kPa, Fig. 5a. However, the components of our compressed gas delivery system for pneumatic actuation were rated only to 110 kPa. Thus, we were only able to test our pneumatic valves up to this value, which is much lower than the burst pressure.

An *ex situ* bonded three-layer PMMA/eCOC/PMMA pneumatic-actuated valve was able to withstand the full range of closing pressures tested (5 to 110 kPa) without delamination (Fig. 5b). The valve closing pressure and fluid pressure (*i.e.*, leakage pressure) followed a linear trend, where each leakage pressure was 5–10 kPa greater than the closing pressure. The leakage pressure closely resembled the closing pressure because the leakage pressure must overcome the pressure of the gas to push the eCOC back up into the displacement chamber to allow the fluid to start flowing. The small difference between closing pressure and leakage pressure is likely due to the limitation of resolution on the non-digital gas tank regulator. At 110 kPa closing gas pressure, the leakage pressure was determined to be 124 kPa, meaning below this pressure no valve delamination or leakage was observed.

Additional tests were run on PMMA/eCOC/PMMA valves to characterize their potential use in integrated microfluidic systems. To test inter-valve variability, tests were performed on 3 different three-layer test valves. Closing pressure *vs.* leakage pressure variance can be seen in Fig. 5c. Comparing closely two closing pressures of 25 and 35 kPa, PMMA/eCOC/PMMA test valves consistently demonstrated leakage pressures of 26.9 ± 3.3 kPa (*n* = 3) and 39.0 ± 1.8 kPa (*n* = 3), respectively. High reproducibility was seen between separate PMMA/eCOC/PMMA test valves, lending itself well to mass production of integrated systems demanding tight tolerances that are demanded by IVDs. Finally, PMMA/eCOC/PMMA valves were actuated 20 times using 25 kPa closing pressure reaching a consistent leakage pressure of 26.9 ± 1.7 kPa with an RSD of 6.3% at 20 μL min^−1^ as can be seen in Fig. 5d. PMMA/eCOC/PMMA valves can reliably seal below their leakage pressure and there was no delamination caused by repeated application of −50 kPa opening pressure even after 20 actuations. For comparison COC/eCOC/COC valves delaminated after 5 actuations.

These valves can be closed with the appropriate closing pressure based on application and known fluidic pressure needed for integrated systems. Similar to mechanical actuation, these valves withstood >20 actuations owing to the high elongation at break of eCOC and sufficient bonding strength between eCOC and the PMMA thermoplastic following *ex situ* bonding and the addition of a pneumatic layer.

### 3.5. Integrated system processing using eCOC valves

The SMART-Chip has been previously reported for the isolation and immunophenotyping of circulating tumor cells (CTCs) from colorectal cancer and pancreatic ductal adenocarcinoma patients' blood samples.^14^ The reported SMART-Chip consisted of a PMMA motherboard containing 11 pneumatic valves with fluidic channels and interconnects used to fluidically connect three modules: a cell isolation module, a cell counting module, and a cell staining and imaging module. The cell isolation chip was able to isolate CTCs in high purity from blood samples (0–3 white blood cells per 2 mL of blood) while the immunophenotyping module was able to retain epithelial cancer cells at an efficiency of 98% to allow for staining and subsequent imaging.^14^ Placing the cell isolation chip on the motherboard with the counting and imaging modules allowed the entire workflow to be automated reducing the chance of contamination or sample loss. Additionally, reduced processing time from 8 h for benchtop assays for isolation and staining of CTCs or CLCs performed by trained operators^88,89^ to <4 h for the modular system was found. PDMS was used as the membrane layer but would fail at ∼10 actuations when used within the integrated system. As we reported above, PMMA to eCOC three-layer valves could operate without failure for >20 actuations and could hold to ∼400 kPa before failure (see Fig. 5a). In comparison, single PMMA/PDMS/PMMA valves were only tested up to 60 kPa forward pressure and 35 kPa closing pressure.^14^

For our system demonstrator, we used the SMART-Chip with eCOC valves to detect MRD by searching for CLCs in a peripheral blood sample and phenotypically identifying the CLCs. There are two subtypes of ALL: B-ALL, which originates in the bone marrow and corresponds to ∼85% of the patient population; and T-cell ALL (T-ALL), which is primarily thymic in origin and affects 15% of the ALL patient population.^90^ The primary cause of death in B/T-ALL patients is due to disease relapse. Therefore, monitoring MRD is considered the most powerful predictor of outcome. Despite significant improvements in the overall survival of children with ALL, ∼20% of B-ALL and 85% T-ALL patients will experience relapse with poor outcomes.^91^ The benefit of pinpointing a patient's MRD and relapse can provide earlier therapeutic intervention with better outcome.

The current standard-of-care for MRD testing uses multi-parameter flow cytometry (MFC), typically from a highly invasive BMA that is not performed frequently due to the invasive nature of the procedure.^92^ We envision an automated IVD test that could potentially be performed as a part of the standard of care in a broader range of clinical testing environments as opposed to MFC of BMAs, which require highly trained medical staff and limit the frequency of testing. The microfluidic MRD assay using the SMART-Chip can be performed for B-ALL patients to either frequently track efficacy of treatment during induction and consolidation or continuously monitor remission during maintenance therapy as well as potentially identify relapse from MRD. The system reported herein used antibodies immobilized to the microfluidic chip surface to affinity select CD19(+) cells from peripheral blood and phenotypically identified CLCs using a staining panel to detect MRD. MRD can then be tracked during post-treatment to determine potential onset of acute disease relapse and also performed frequently as it is considered minimally invasive.

**Fig. 6:**
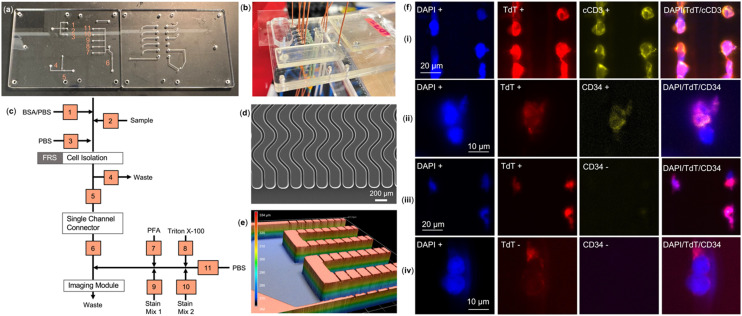
(a) Image of fluidic and pneumatic layers of the PMMA SMART-Chip with 11 valves labeled on the fluidic layer. (b) Fully assembled SMART-Chip with 3 modules: cell selection device, jumper module, and imaging module. (c) Valve state diagram of system showing how the 11 valves control various reagents flow to each of the modules, (FRS = flow rate sensitive, PFA = 3% paraformaldehyde solution, stain mix 1 = antibody staining (TdT and cCD3 or CD34), and stain mix 2 = DAPI). (d) SEM of sinusoidal channels in the cell selection module; (e) Keyence optical profilometer *z*-depth image of imaging module. (f) CD7+ MOLT-3 cells or CD19+ cells from a B-ALL blood sample were captured on an anti-CD7 or anti-CD19 modified PC-linker cell isolation module mounted onto the SMART-Chip system seen in (b). Following capture, cells were exposed to 400–450 nm light and released into the imaging module mounted on the motherboard where immunophenotyping was performed: (i): DAPI, TdT, and cCD3 signals of MOLT-3 cells shown trapped at pores; (ii) DAPI, TdT, CD34 signals from a normal and a CLC from B-ALL clinical blood sample; (iii) three CLCs from a B-ALL clinical samples; (iv) two normal B-cells from a B-ALL clinical sample. Exposure times: DAPI (20 ms),), Cy5 (3000 ms), Cy7 (5000 ms).

Fig. 6a shows the location of the 11 valves on the fluidic layer of the motherboard. The motherboard allowed for introduction of eight reagents *via* input ports and contained two waste outlets. It also contained several features that allowed alignment of the fluidic and pneumatic layers during assembly. Fig. 6b shows the fully assembled PMMA/eCOC/PMMA motherboard with: (i) cell isolation module; (ii) jumper module; and (iii) imaging module. For this application, only the cell isolation module (Fig. 6d) and the cell staining/imaging module (Fig. 6e) were employed. The valve state diagram for processing the sample on the SMART-Chip can be seen in Fig. 6c where each valve corresponds to squares in the diagram. The solenoid controller was programmed to open/close each valve necessary for each step in the workflow. The impedance sensing module was not included in this application because the affinity isolation of CLCs using anti-CD19 antibodies selects both normal B-cells and CLCs and as such, following release, the impedance sensor for enumeration serves no useful purpose. Thus, enumeration depends on results secured *via* immunophenotyping of the selected cells.

We first used the MOLT-3 cell line to show successful isolation and immunophenotyping of cells on the SMART-Chip using the necessary monoclonal antibody, mAb (CD7; indicative of T-ALL) and staining panel. The eCOC pneumatic valves were used to direct the fluid during processing as seen in the workflow shown in Fig. 6c. The workflow consisted of cell suspension infusion through the cell isolation chip (valves 1–5), and upon photo-release from the surface, the selected cells were directed from the cell isolation module to the imaging module (valves 3–6). The immunophenotyping procedure consisted of PFA and Triton X-100 treatments and staining for the presence of antigen and nuclear markers (*i.e.*, anti-cCD3, anti-TdT antibodies and DAPI,) performed by opening/closing the appropriate eCOC valves (valves 7–11). As expected, MOLT-3 cells showed expression of TdT and cCD3 (cytoplasmic CD3) and the presence of the nuclear DAPI stain (see Fig. 6f(i)). Additionally, a model cell line for B-ALL, SUP-B15, was also evaluated using the SMART-Chip and efficiency of capture and trapping of the cells can be found in the ESI† (Fig. S7).

We next processed a B-ALL patient blood sample (∼1 mL) through the SMART-Chip with anti-CD19 mAbs attached *via* a PC-linker to the walls of the cell isolation module. Employing the same workflow described above, in 1 mL of blood, 441 DAPI(+) cells and 26 DAPI(+)/TdT(+) cells were detected. TdT(+) cells were identified as CLCs and considering a mononucleated white blood cell count of 1.76 × 10^6^ per mL, the patient's MRD was calculated to be 0.0015%, or 1 leukemia cell in 70 000 mononucleated cells. The advantage of the integrated system is a 7× improvement in sensitivity was found. At the time of testing (end of maintenance therapy, 11 months post-diagnosis), this patient was considered to be in remission as determined by a corresponding MFC of a BMA. Several images of both healthy and CLCs can be seen in Fig. 6f(ii–iv). As seen in Fig. 6f(ii), two cells were visible where one cell can be considered a normal B-cell (DAPI(+)/TdT(−)/CD34(−)), and the other a CLC (DAPI(+)/TdT(+)/CD34(+)). However, it should be noted that both cells express CD19 and this is an important differentiator for CLCs compared to CTCs – the selection mAb selects normal and diseased cells that is not typically seen using anti-EpCAM mAbs. Three CLCs can be seen in Fig. 6f(iii) with a TdT(+) and CD34(−) phenotype. Finally, two normal B cells (DAPI(+)/TdT(−)) can be seen in Fig. 6f(iv) that were isolated by the selection chip.

The MRD threshold is determined by the sensitivity of the analytical technique. For MFC, the sensitivity of a 3 or 4 color system is 0.01% or 10^−4^, meaning MFC can identify 1 leukemia cell among 10 000 mononucleated cells.^93^ Relying on diagnosis based on detection of 1 leukemia cell is not reliable, therefore, it means that to detect 10 leukemia cells, the MFC must interrogate 1 × 10^5^ cells. Even 5 and 6 color MFC are not sensitive to consistently and reliably detect below 10^−4^.^93^ For this reason, the MRD threshold used by the Children's Oncology Group for risk stratification using MFC stands at 0.01%.^94^

Presented herein data secured with the SMART-Chip for MRD detection in B-ALL confirmed our earlier findings in other type of leukemias, such as acute myeloid leukemia (AML) and plasma cell disorders (*i.e.*, multiple myeloma, MGUS, SMM, and AMM) that the microfluidic chip-based MRD detection exceeds the MFC-MRD threshold.^88,89^ In these publications, we demonstrated the utility of our cell selection device for the high sensitivity isolation of cells directly from peripheral blood, eliminating the need for BMA. Owing to the ability of frequent MRD testing and observing changes in CLC burden in patients undergoing treatment, we presented evidence in AML that the assay identified signs of impending relapse >2 months earlier than BMA-based tests.^88^

During sample processing, neither eCOC valves nor microfluidic chips showed signs of delamination or failure, thus demonstrating eCOC's potential to be integrated into microfluidic systems. The integrated system used here for MRD testing provides benefits for frequent minimally invasive testing to pinpoint when a patient's MRD is suggestive of relapse. Such information can provide earlier intervention with potentially better pediatric patient outcome.

## 4. Conclusion

Mixed-scale integrated fluidic systems require valving to efficiently and autonomously deliver reagents/sample, control fluid flow, and fluidically connect devices as part of their operation. Typical valving techniques for microfluidic systems, which can produce relatively high pressures, require laborious fabrication/assembly techniques to allow for successful operation at the necessary fluidic pressures. Here we presented and characterized eCOC membranes and two simple assembly methods that provide high holding pressures in two different valving modalities using various thermoplastic substrates. In addition, eCOC used for the valving membrane can be extruded into various film thicknesses and simply bonded to a variety of thermoplastics that can be mass produced using injection molding or extrusion at low cost and high process yield rates making the necessary system commensurate with IVD applications.

When designing integrated systems, cost of production, simplicity in system assembly, low system operational failure rates, high process yield rates, and ease of use are important factors for the potential use of the system irrespective of application. Mechanically actuated membrane valves use a physical force to push an elastomer onto a valve seat, thus closing that channel to fluid flow. Mechanically actuated valves allow for the valving system to be directly integrated into the system with low requirements for the external hardware, which can be located off-chip. Thus, the system can be designed for single use operation and the instrument hardware can be situated off-chip to reduce assay cost. Mechanical actuation demonstrated in this study uses small (1′′ tall × 0.5′′ diameter) and low-cost solenoids (<$20) that can be easily implemented into a small footprint instrument. On the contrary, pneumatic actuation employed gas pressure to push an elastomer onto the valve seat and vacuum to pull the elastomer off the valve seat in some cases. Compared to mechanical actuation, pneumatic actuation requires a larger overall footprint and more external components. However, eCOC can be used with either valve actuation method as was demonstrated by implementing eCOC as the membrane layer.

The two-layer valve composed of PETG with eCOC held up to 350 kPa fluidic pressure, and the three-layer valve composed of PMMA with eCOC held up to 400 kPa. Typical PDMS and glass valve assemblies report holding to 75–200 kPa.^26,27,48^ Although alternative elastomers have been proposed, they generally require complex fabrication and assembly methods. Eliminating the need for added heat and pressure for assembly, or additional adhesives/solvents makes the use of eCOC with a thermoplastic substrate a low-cost process, highly reproducible, and an excellent process yield rate (∼100%). As shown, eCOC valves can be actuated ∼50 times without sign of wear, mainly resulting from its large elongation at break (>500%). eCOC's surface can be activated with UV/O_3_ and demonstrates hydrophilic stability.

*In situ* bonding of eCOC to PETG and PC could easily be implemented into large-scale production lines for a variety of applications. In addition, the lack of need for added adhesives or solvents, or elevated heat exasperates the simplicity and low-cost associated with device assembly. Finally, eCOC can be continuously extruded in roll form and laminated or rolled onto microdevices in large scale then exposed to UV/O_3_ to further increase device production rate; this is known as roll-to-roll production.^95^ However, one issue that should be noted is that the thermal properties of PC can be considered superior to PETG due to its higher *T*_g_. Thus, in cases where thermal reactions are required for the system such as PCRs, PC may be the material of choice.

Finally, the eCOC membrane valves were successfully integrated into a modular system to both select and immunophenotype CLCs selected from the blood sample of a patient diagnosed with B-ALL. The CLCs could be successfully enumerated using a panel of markers to differentiate between normal B-cells and those with leukemic properties. This modular system also served to demonstrate the versatility of eCOC in the use of pneumatic valves with PMMA as the fluidic and pneumatic layers.

## Data availability

All data associated with this manuscript is detailed in the form of figures and tables included in the manuscript as well as material shown in the ESI.† The data presented in all figures and tables can be made available to the readership upon request to the corresponding author of this manuscript. Information regarding the identity of human blood sample donors cannot be provided due to confidentiality reasons.

## Author contributions

K. C.: investigation, writing – original draft, writing – revising and editing. I. M. F.: investigation, writing – original draft. M. L. H.: conceptualization, methodology, writing – original draft, writing – revising and editing. B. S.: investigation. N. L.: investigation. P. H.: resources. J. H. N.: resources. F. S.: resources. J. V.: resources. K. J. A.: resources. M. A. W.: conceptualization, visualization, writing – original draft, writing – revising and editing, funding acquisition. S. A. S.: conceptualization, supervision, funding acquisition, writing – revising and editing.

## Conflicts of interest

There are no conflicts to declare.

## Supplementary Material

LC-024-D4LC00501E-s001
