# Supplementary material for: Novel thermoplastic microvalves based on an elastomeric cyclic olefin copolymer
Source: Lab Chip. 2024 Aug 13;24(18):4422–39. doi: 10.1039/d4lc00501e (PMC11339931; doi:10.1039/d4lc00501e)
Supplement: LC-024-D4LC00501E-s001 [file LC-024-D4LC00501E-s001.pdf]

Supplementary Information (SI) for Lab on a Chip.
This journal is © The Royal Society of Chemistry 2024

Supporting Information

# Novel Thermoplastic Microvalves Based on an Elastomeric Cyclic Olefin Copolymer

Katie Childers,[1,2] Ian M. Freed,[2,3] Mateusz L. Hupert,[4] Benjamin Shaw,[2,5] Noah Larsen,[2,6] Paul Herring,[7] Jeanne H. Norton,[7] Farhad Shiri,[2,3] Judy Vun,[8] Keith J. August,[8] Małgorzata A. Witek,[2,3] Steven A. Soper[1,2,3,9,10*]

[1]Bioengineering Program, The University of Kansas, Lawrence, KS 66045
[2]Center of Biomodular Multiscale Systems for Precision Medicine
[3]Department of Chemistry, The University of Kansas, Lawrence, KS 66045
[4]BioFluidica Inc., San Diego, CA 92121, USA.
[5]Department of Chemical Engineering, The University of Kansas, Lawrence, KS 66045
[6]Department of Engineering Physics, The University of Kansas, Lawrence, KS 66045
[7]Department of Plastics Engineering Technology, Pittsburg State University, Pittsburg, KS 66762
[8]Children's Mercy Hospital, Kansas City, MO 64108, USA
[9]Department of Mechanical Engineering, The University of Kansas, Lawrence, KS 66045
[10]KU Cancer Center, University of Kansas Medical Center, Kansas City, KS 66160

*Author to whom correspondence should be addressed.

**Milling Parameters**

Individual test valve structures were milled using a desktop CNC milling machine (Nomad3, Carbine 3D, Torrance, CA). Three milling bits (1/16", 1/32", and 500 µm) were utilized to mill the various microscale features for both the pneumatic and mechanical actuation test valves. Microchannels were milled using the 1/32" bit at a plunge rate of 200 mm/min, a feed rate of 250 mm/min, and 3,500 RPM. The inlet and outlet for PEEK tubing connection were milled with the 1/32" bit using the same milling conditions. The 1/32" bit was used so that the inlet/outlet would be of equal diameter to that of the PEEK tubing with an outer diameter of 1/32". The epoxy used to attach the tubing was thoroughly and vigorously mixed by hand and allowed to sit for 2 – 3 min to increase in viscosity so that the epoxy would not flow into the fluidic microchannel. A small bead of epoxy was applied to the end of the tubing before being inserted into the inlet and outlet hole. The 500 µm bit was used to make the through hole in the center of the mechanical actuation test valves at a plunge rate of 150 mm/min, a feed rate of 250 mm/min, and bit spin speed of 9,000 RPM. The solenoid attachment holes (diameter = 2 mm) were milled using the 1/16" bit at a plunge rate of 250 mm/min, a feed rate of 350 mm/min, and 9,000 RPM. Finally, the 1/16" bit was used to mill out the final test valves using a plunge rate of 450 mm/min, a feed rate of 1,500 mm/min, and 15,000 RPM.

**Cell Isolation Module and Imaging Module Preparation**

The PC linker and mAb immobilization to a thermoplastic surface has been previously reported by our group.[1] Briefly, COP cell selection modules were UV/$O_3$ activated (22 mW/cm$^2$, 13 min) and incubated with an EDC (20 mg/mL) / NHS (2 mg/mL) solution for 25 min at room temperature in dry ACN. The solution was removed from the module by introducing air, and then the PC linker (1 mM) dissolved in dry ACN and TEA (2 mM) was infused into the module. After 2 h of incubation at room temperature, the solution was replaced by passing air through the module. Then, 100 mM Tris (pH 7.4) was introduced into the module and incubated for 30 min at room temperature. After incubation, the EDC/NHS solution was replaced with the mAb (anti-CD7 or anti-CD19, 1 mg/mL) dissolved in PBS (pH 7.4) and incubated overnight at 4°C. Prior to sample infusion, the module was rinsed with 2 mL of 0.5% BSA/PBS at 50 µL/min to remove unbound mAbs and to minimize non-specific binding.

Imaging modules made from PDMS (Sylgard-184, Dow Corning) were cast against a SU-8 positive relief with the resulting PDMS structures consisting of traps and microchannels.[2] A 10:1 ratio of base:curing agent of PDMS was used and was mixed in a large weigh-boat for 5 min prior to pouring onto the relief. After pouring the PDMS onto the relief structures, it was placed into a

vacuum chamber for 1 hr. The PDMS was then baked in a convection oven at 75°C for 5 hr. After curing, individual devices were cut from the relief and inlet/outlet holes were punched using a 1.25 mm biopsy punch, then subsequently washed with 10% Micro-90 solution with light abrasion (toothbrush under running water), and finally rinsed with IPA then dried using compressed air. Devices were then cleaned and dried in a 60°C oven for 20 min prior to bonding. Bonding was performed using glass cover plates that were first cleaned with IPA. To bond, both the cleaned device and cover plate were placed into an $O_2$ plasma chamber (90 W, 25 sccm $O_2$) for 1 min (Diener Electronic, GmbH & Co KG; Germany) — after 1 min exposure, the cover plate was lightly pressed against the device and irreversible bonding was complete.

## Hydrophobic Recovery of PDMS

Sessile water contact angle (WCA) measurements were performed on PDMS (Standard Rubber Products Co., HT6240) and eCOC (TOPAS Polyplastics) after various times following $O_2$ plasma activation (1 min, 20 sscm $O_2$ gas flow, 50 W). Within 72 h, PDMS had a WCA of 96° ±5.4°, but the eCOC remained stable with an average of 73° ±2.5° over the same time period as well as much longer time periods (see **Fig. S1**). PDMS hydrophilic recovery is partially due to the movement of low molecular weight species showing facile movement into the bulk of the polymer, while thermoplastics like eCOC have larger fragments in comparison following oxidation and are less apt to diffuse back into the bulk of the polymer.[3-5]

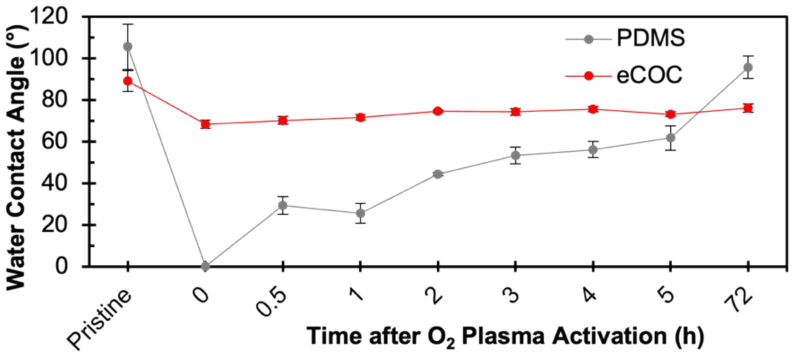


**Figure S1**. WCA measurements of PDMS and eCOC before and after surface activation (1 min $O_2$ plasma at 20 sscm $O_2$ gas flow and 50 W) over the course of 72 h.

## WCA of Thermoplastics Exposed to UV/$O_3$ Radiation

Sessile WCA measurements were performed on pristine COC, COP, PC, PETG, and PMMA. UV/$O_3$ activation was performed through eCOC and quartz plates (1 mm thickness) placed between the eCOC and the appropriate plastic (see **Fig. S2a**), as well as exposing the plastic surface directly (**Fig. S2b**). The graphs in **Fig. S2c** shows the resulting WCAs before and after

$UV/O_3$ exposure and with/without the eCOC/quartz plate. Interestingly, PETG showed a significant reduction in its WCA whether the eCOC/quartz cover was in place or not. A more thorough description of this data can be found in the main text.

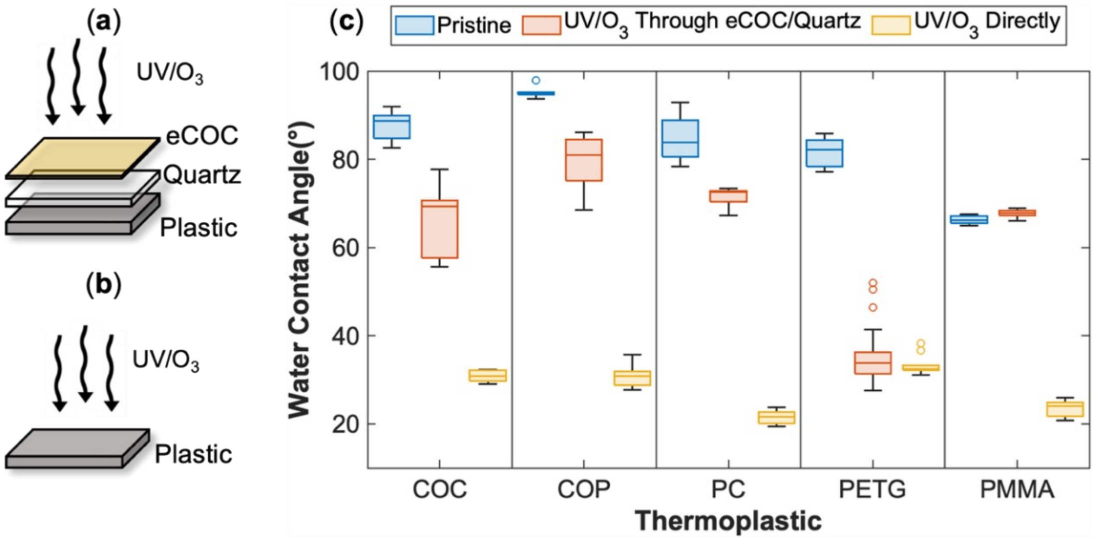


**Figure S2**. (**a**) Diagram of how the tested plastic was exposed to $UV/O_3$ by first passing through a layer of eCOC and quartz. (**b**) Diagram of a particular plastic directly exposed to $UV/O_3$. (**c**) WCA measurements for pristine COC, COP, PC, PETG, and PMMA and after each plastic was exposed to $UV/O_3$ using the arrangement shown in (**a**) or (**b**).

## Surface Characterization of eCOC films

All eCOC films became more hydrophilic after $UV/O_3$ exposure as noted by decreases in their water contact angles. There was no significant difference in the water contact angles between the supplier films (TOPAS Polyplastics and Roehm) or between the extruded films (all were in the range of 60 – 70°). In addition, ATR-FTIR measurements did not show any noticeable difference in FTIR bands following 10 min $UV/O_3$ activation between different films.

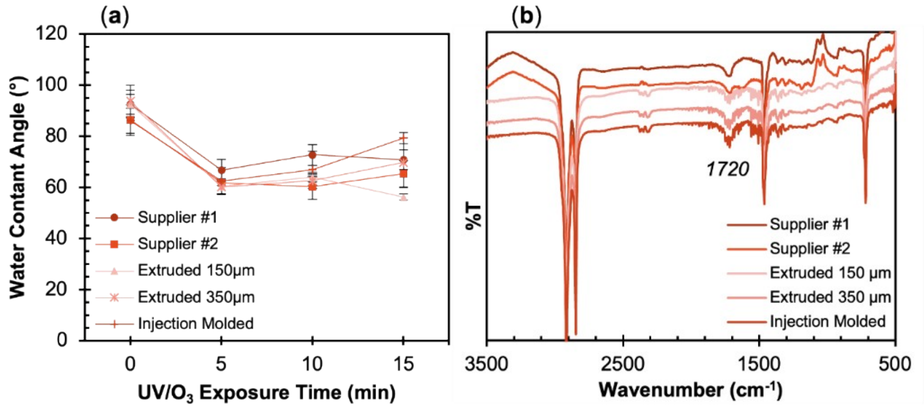


**Figure S3**. (**a**) Water contact angle results comparing supplier-provided eCOC films (Supplier #1 – TOPAS Polyplastics, Supplier #2 – Roehm), cast film extruded films (150 µm and 350 µm thick), and one eCOC sample that was injected molded. Water contact angle measurements were taken following 5-, 10-, and 15-min $UV/O_3$ activation times. (**b**) ATR-FTIR data comparing various eCOC films after 10 min $UV/O_3$ activation, they all show a peak around 1720 $cm^{-1}$ indicative of a carbonyl group (*i.e.*, carboxy, aldehyde or ketone).

## UV-Vis Spectra of Pristine eCOC

UV-Vis spectra were taken for pristine eCOC at different thicknesses (130, 150, 280, and 350 µm) as compared to **Fig. 3**(**a**) in the main manuscript, which were taken following UV/$O_3$ activation. As the thickness of the eCOC increased, the UV transmittance decreased when monitored at 254 nm, which is due to an increase in the effective pathlength. This was substantiated by the fact that careful inspection of the spectra shown in **Fig. S4** did not show any new bands in the spectra as the plastic thickness was changed.

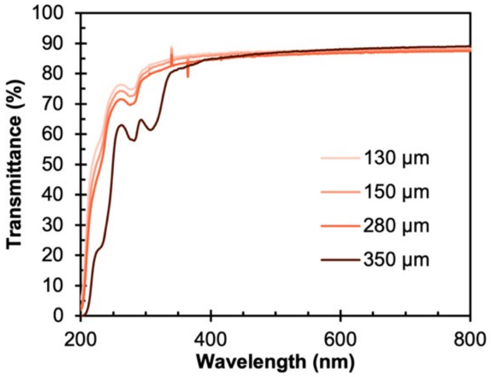


**Figure S4.** UV-visible spectrum showing transmittance for pristine eCOC of various thicknesses (130, 150, 280, and 350 µm).

## ATR-FTIR of PETG

ATR-FTIR results did not show any significant differences between pristine and 10 min UV/$O_3$ activated PETG, because PETG contains many functional groups in its pristine state.

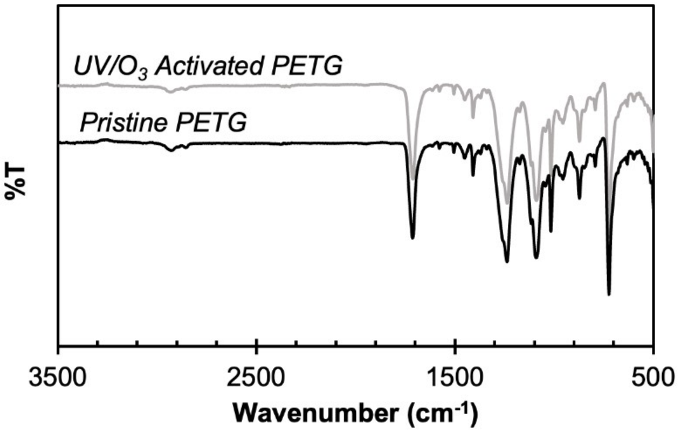


**Figure S5.** ATR-FTIR traces for pristine and UV/$O_3$ activated PETG.

## Photodegradation Mechanism of PETG

Grossetete *et al.* studied the photodegradation of PETG under both polychromatic irradiation and monochromatic irradiation.[6] The PETG samples were artificially weathered for 20 – 150 h and changes to the surface chemistry were observed via UV, emission, and IR spectra. They found that the aromatic chromophores in PETG strongly absorb radiation <350 nm inducing a photolytic process as proposed in **Fig. S6**. Polychromatic and monochromatic radiation resulted in the creation of similar photoproducts, but with a difference in the distribution profile of the resulting photoproducts. Photoproducts produced with 254 nm light remain in the first 5 µm of the substrate surface with longer wavelengths reaching 50 µm into the substrate. We hypothesize that it is these photoproducts, including a number of different highly reactive radicals, that can react with eCOC polymer chains that also generate photolytically induced radicals albeit at a lower abundancy generating cross linked polymer chains that facilitate strong bonding without requiring heat. The heating induces polymer chain motion that allows cover plate/substrate bonding in other cases.

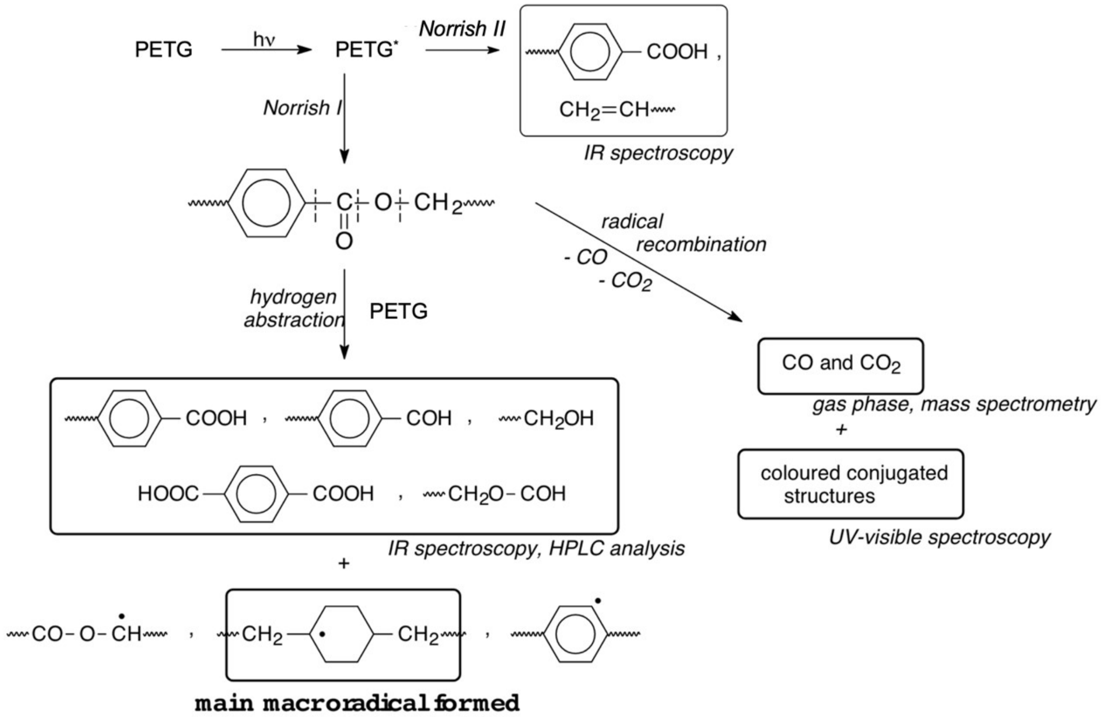


**Figure S6.** Scheme of chemical reactions of PETG caused by UV/$O_3$ exposure [adapted from Grossetête *et al.]*.[6]

## SUP-B15 Cell Isolation using SMART-Chip

Before processing the clinical blood sample, we evaluated the SMART-Chip using SUP-B15 cell line, which is a model for B-ALL circulating leukemia cells. The recovery of these cells was found to be ~57 ±12% at volume flow rate of whole blood equal to 25 µL/min with 87% release efficiency

from the cell isolation chip. Spiked SUP-B15 into 500 µL PBS were retained on the imaging chip at an efficiency of 85.4%. The staining protocol for the SUP-B15 cell line followed these steps: (i) Staining for surface antigens (CD10, CD34; 30 min); (ii) fixation with 2% buffered PFA (10 min); (iii) permeabilization with 0.1% Triton X-100 (2 min); and (iv) staining for TdT and DAPI (30 min). We successfully implemented the staining protocol to immunophenotype SUP-B15 cells trapped within the imaging module as shown in **Fig. S7**. SUP-B15 cells are nucleated and should be positive for TdT and CD10 as shown in **Fig. S7**.

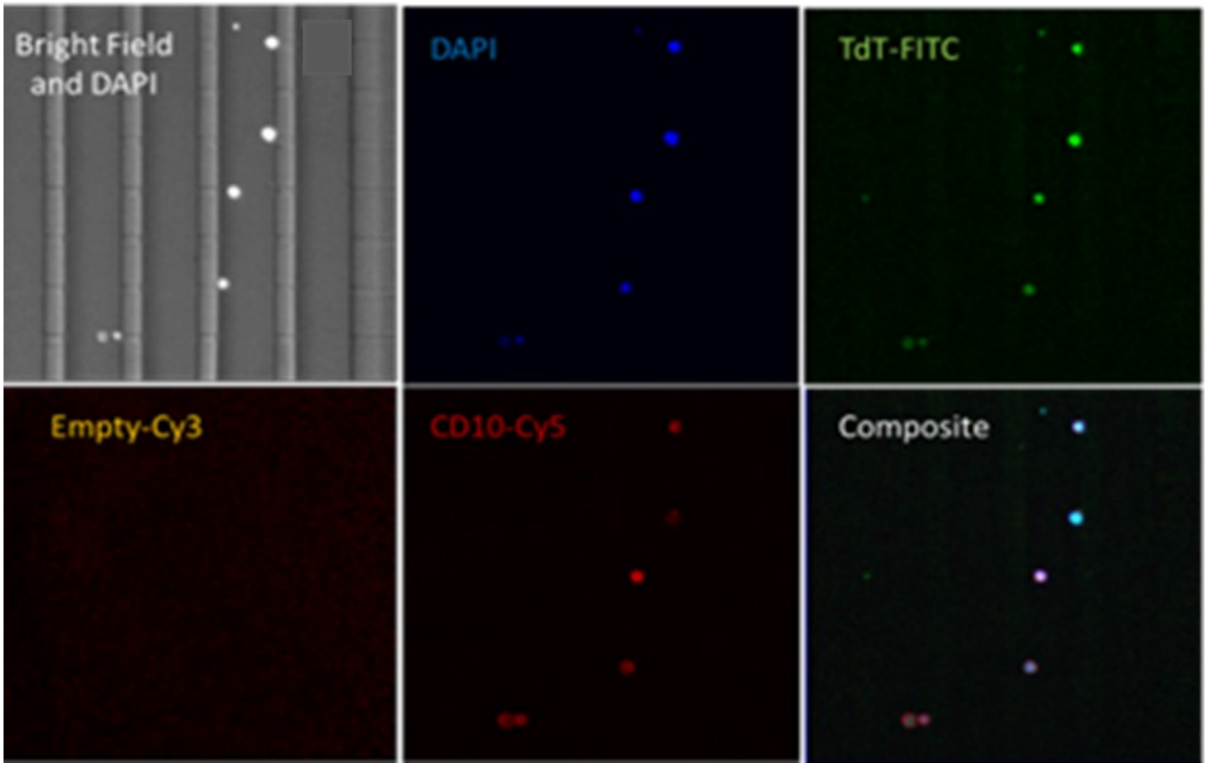


**Figure S7.** SUP-B15 cell staining performed on the SMART-Chip. DAPI, TdT, and CD10 signals of SUP-B15 cells trapped at the containment pores of the imaging chip.

## References


1. T. N. Pahattuge, J. M. Jackson, R. Digamber, H. Wijerathne, V. Brown, M. A. Witek, C. Perera, R. S. Givens, B. R. Peterson and S. A. Soper, *Chemical Communications*, 2020, **56**, 4098-4101.

2. K. M. Weerakoon-Ratnayake, S. Vaidyanathan, N. Larkey, K. Dathathreya, M. J. Hu, J. Jose, S. Mog, K. August, A. K. Godwin, M. L. Hupert, M. A. Witek and S. A. Soper, *Cells-Basel*, 2020, **9**.

3. D. Bodas and C. Khan-Malek, *Sensor Actuat B-Chem*, 2007, **123**, 368-373.

4. J. M. Jackson, M. A. Witek, M. L. Hupert, C. Brady, S. Pullagurla, J. Kamande, R. D. Aufforth, C. J. Tignanelli, R. J. Torphy, J. Jen Yeh and S. A. Soper, *Lab on a Chip*, 2014, **14**, 106-117.

5. S. Vaidyanathan, H. Wijerathne, S. S. T. Gamage, F. Shiri, Z. Zhao, J. Choi, S. Park, M. A. Witek, C. McKinney, M. Verber, A. R. Hall, K. Childers, T. McNickle, S. Mog, E. Yeh, A. K. Godwin and S. A. Soper, *Anal Chem*, 2023, **95**, 9892-9900.

6. T. Grossetête, A. Rivaton, J. L. Gardette, C. E. Hoyle, M. Ziemer, D. R. Fagerburg and H. Clauberg, *Polymer*, 2000, **41**, 3541-3554.
